# Functional and structural analysis of a cyclization domain in a cyclic β-1,2-glucan synthase

**DOI:** 10.1007/s00253-024-13013-9

**Published:** 2024-02-01

**Authors:** Nobukiyo Tanaka, Ryotaro Saito, Kaito Kobayashi, Hiroyuki Nakai, Shogo Kamo, Kouji Kuramochi, Hayao Taguchi, Masahiro Nakajima, Tomoko Masaike

**Affiliations:** 1 Department of Applied Biological Science, Faculty of Science and Technology, Tokyo University of Science, 2641 Yamazaki, Noda, Chiba 278-8510 Japan; 2 Artificial Intelligence Research Center, National Institute of Advanced Industrial Science and Technology (AIST), 2-4-7 Aomi, Koto-Ku, Tokyo 135-0064 Japan; 3 Faculty of Agriculture, Niigata University, Niigata, 950-2181 Japan

**Keywords:** Cyclic β-1,2-glucan, β-1,2-Glucan, Glycoside hydrolase family, Transglycosylation

## Abstract

**Abstract:**

Cyclic β-1,2-glucan synthase (CGS) is a key enzyme in production of cyclic β-1,2-glucans (CβGs) which are involved in bacterial infection or symbiosis to host organisms. Nevertheless, a mechanism of cyclization, the final step in the CGS reaction, has not been fully understood. Here we performed functional and structural analyses of the cyclization domain of CGS alone from *Thermoanaerobacter italicus* (TiCGS_Cy_). We first found that β-glucosidase-resistant compounds are produced by TiCGS_Cy_ with linear β-1,2-glucans as substrates. The ^1^H-NMR analysis revealed that these products are CβGs. Next, action pattern analyses using β-1,2-glucooligosaccharides revealed a unique reaction pattern: exclusive transglycosylation without hydrolysis and a hexasaccharide being the minimum length of the substrate. These analyses also showed that longer substrate β-1,2-glucooligosaccharides are preferred, being consistent with the fact that CGSs generally produce CβGs with degrees of polymerization of around 20. Finally, the overall structure of the cyclization domain of TiCGS_Cy_ was found to be similar to those of β-1,2-glucanases in phylogenetically different groups. Meanwhile, the identified catalytic residues indicated clear differences in the reaction pathways between these enzymes. Overall, we propose a novel reaction mechanism of TiCGS_Cy_. Thus, the present group of CGSs defines a new glycoside hydrolase family, GH189.

**Key points:**

*• It was clearly evidenced that cyclization domain alone produces cyclic β-1,2-glucans*.

*• The domain exclusively catalyzes transglycosylation without hydrolysis*.

*• The present catalytic domain defines as a new glycoside hydrolase family 189*.

**Graphical Abstract:**

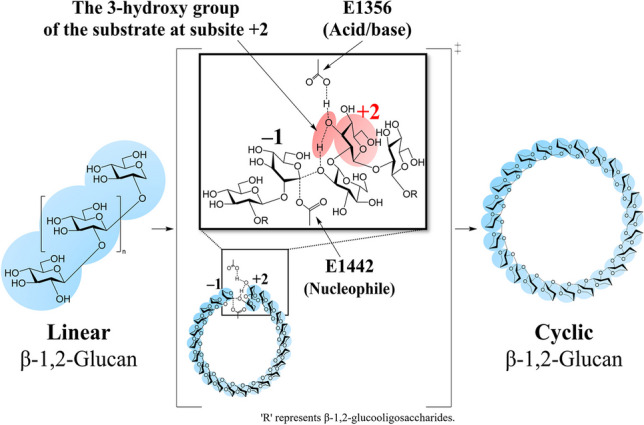

**Supplementary Information:**

The online version contains supplementary material available at 10.1007/s00253-024-13013-9.

## Introduction

β-1,2-Glucan is a polysaccharide comprising glucose units linked by β-1,2-glucosidic bonds. In nature, cyclic forms are mainly found in bacteria such as *Agrobacterium tumefaciens*, *Brucella abortus*, and *Ensifer meliloti* (formerly *Rhizobium meliloti* and *Sinorhizobium meliloti*) (Dell et al. 1983; Bundle et al. 1988; Koizumi et al. 1984). Cyclic β-1,2-glucans (CβGs) play important roles in interactions between organisms such as infection of *B. abortus* and symbiosis of *E. meliloti* (Breedveld and Miller 1994; Haag et al. 2010; Dylan et al. 1990). Genes encoding enzymes responsible for CβG biosynthesis were identified from three microorganisms independently. These genes were found to encode cyclic β-1,2-glucan synthases (CGSs) homologous to each other (Zorreguieta and Ugalde 1986; Castro et al. 1996; Iannino et al. 1998). Among them, CGS from *B. abortus* (BaCGS) produces CβGs with degrees of polymerization (DPs) around 20 and is most extensively characterized (Bundle et al. 1988; Guidolin et al. 2009). BaCGS is composed of three regions responsible for the following steps: initiation (covalent bonding of glucose to CGS), elongation of linear β-1,2-glucan (LβGs) chains, adjustment of chain lengths of the glucans, and cyclization of the glucans by transglycosylation (Fig. S1) (Guidolin et al. 2009). The initiation and the elongation steps are carried out in the N-terminal domain classified into glycosyltransferase (GT) family 84 based on amino acid sequence homology by carbohydrate-active enzyme database (CAZy) (Coutinho et al. 2003; Drula et al. 2022; Guidolin et al. 2009). The C-terminal glycoside hydrolase (GH) family 94 domain adjusts the chain lengths of the elongated glucans (Ciocchini et al. 2006; Guidolin et al. 2009). Although the domain in the middle region is known to be involved in cyclization of the linear glucans of the optimum lengths (hereafter, this domain is called cyclization domain), a detailed reaction mechanism has not been unveiled.

Apart from the context of synthetic enzymes described above, β-1,2-glucan-degrading enzymes have been investigated (Abe et al. 2017; Tanaka et al. 2019; Nakajima 2023). Owing to establishment of a large-scale preparation of LβGs by using a 1,2-β-oligoglucan phosphorylase (SOGP) found in 2014 (Nakajima et al. 2014; Abe et al. 2015), prokaryotic and eukaryotic *endo*-β-1,2-glucanases (SGLs) that produce β-1,2-glucooligosaccharides (Sop_n_s, n is DP) from β-1,2-glucans were explored. Consequently, a bacterial SGL from *Chitinophaga pinensis* (CpSGL) and a fungal SGL from *Talaromyces funiculosus* (TfSGL) were sequentially identified for the first time, leading to creation of new families (GH144 and GH162), respectively (Abe et al. 2017; Tanaka et al. 2019). Subsequently, functions and structures of several β-1,2-glucan-associated enzymes including the ones that are given new EC numbers were reported (Nakajima et al. 2016, 2017; Shimizu et al. 2018; Kobayashi et al. 2022). In addition, another SGL which belongs to neither GH144 nor GH162 has been identified from *Escherichia coli* very recently, leading to foundation of a new family, GH186 (Motouchi et al. 2023).

Interestingly, both CpSGL and TfSGL possess single (α/α)_6_-domains with similar overall structures although they belong to different families (Abe et al. 2017; Tanaka et al. 2019). Therefore, PSI-BLAST search was performed using CpSGL and TfSGL as queries to find further homologs with evolutional relationships. As a result, the cyclization domains of CGSs (CGS_Cy_s) came up although the domains do not belong to any GH families. In the previous study, we showed that the general acid, one of the two catalytic residues, of TfSGL (GH162) exhibits a unique catalytic mechanism that acts via a 3-hydroxy group of the glucose moiety (Tanaka et al. 2019). This mechanism rarely found in the anomer-inverting type is somehow highly conserved among cyclization domains of anomer-retaining CGSs. Therefore, CGSs may share a common mechanism with GH162 beyond inverting/retaining mechanisms. However, TfSGL and CGS_Cy_ are intrinsically different in terms of reaction mechanisms. TfSGL follows the anomer-inverting mechanism in which anomer of substrates changes when products are released, while CGS is considered to follow the anomer-retaining mechanism in that substrates and products share the same anomer (see https://www.cazypedia.org/index.php/Glycoside_hydrolases#Mechanistic_classification for detail) (The CAZypedia Consortium 2018; Davies and Henrissat 1995). Thus, we predicted that CGS_Cy_ follows a noncanonical reaction mechanism. In this study, we subcloned a region encoding the cyclization domain alone from CGS of *Thermoanaerobacter italicus* (TiCGS), a thermophilic bacterium, and explored biochemical functions and tertiary structure of the cyclization domain.

## Materials and methods

### Materials

The genomic DNA of *T. italicus* (DSM9252) was purchased from the National Institute of Technology and Evaluation (NITE, Tokyo, Japan). LβGs with the average DP of 77 (unless otherwise described, average DP of the β-1,2-glucans used in the present study is 77) and Sop_n_s with DP of 2–10 were prepared using SOGP from *Listeria innocua* and CpSGL as described previously (Nakajima et al. 2014; Abe et al. 2015, 2017). CβGs with DPs of 17–24 were kindly donated by Dr. M. Hisamatsu of Mie University (Hisamatsu et al. 1984). Laminarin and carboxymethyl (CM)-cellulose were purchased from Sigma-Aldrich (MO, USA). CM-pachyman, CM-curdlan, lichenan, tamarind xyloglucan, arabinogalactan, arabinan, and polygalacturonic acid were purchased from Neogen (MI, USA).

### Cloning, expression, and purification of TiCGS_Cy_

A middle region (1005–1591 a.a.) of TiCGS (KEGG locus, Thit_1831) (TiCGS_Cy_) was used for cloning (see the “Results” section for details). The gene region was inserted into the pET30a vector (Merck, NJ, USA) according to the manufacturer’s instructions so that histidine-tag derived from the vector is fused at C-terminus.

*E. coli* Rosetta2 (DE3) (Merck) was transformed using the constructed plasmid and cultured at 37 °C in LB medium containing 30 μg/ml kanamycin and 34 μg/ml chloramphenicol. After the optical density of the culture at 660 nm reached 0.8, protein expression was induced using 0.1 mM isopropyl-β-d-1-thiogalactopyranoside at 20 °C overnight. The harvested cells were lysed by sonication in 50 mM Tris–HCl buffer (pH 8.0) containing 150 mM NaCl. The supernatant was collected after centrifugation at 27,700 × *g*. Then the supernatant was filtrated with a 0.45-μm filter (Sartorius, Germany). The sample was loaded onto a HisTrap FF crude column (5 ml; Cytiva, MA, USA) equilibrated with 50 mM Tris–HCl buffer (pH 8.0) containing 150 mM NaCl (buffer A) using an AKTA explorer chromatography system (Cytiva). After unbound proteins were washed out using the same buffer containing 20 mM imidazole, TiCGS_Cy_ was eluted using a linear imidazole concentration gradient (20–300 mM) in buffer A. 2 M ammonium sulfate solution containing 100 mM sodium acetate buffer (pH 5.0) was added to the collected sample to obtain 1 M ammonium sulfate concentration. After unbound proteins were washed out using the 1 M ammonium sulfate containing 100 mM sodium acetate buffer (pH 5.0), TiCGS_Cy_ was eluted using a linear ammonium sulfate concentration gradient (1–0 M) in 100 mM sodium acetate buffer (pH 5.0). The enzyme solution was exchanged with 5 mM sodium acetate buffer (pH 5.0) using Amicon Ultra 10,000 molecular weight cut-off (Merck). The absorbance of the sample at 280 nm was measured using a spectrophotometer V-650 (Jasco, Tokyo, Japan), and the concentration of the enzyme was determined spectrophotometrically at 280 nm using the theoretical molecular mass of TiCGS_Cy_ (69,508 Da) and a molar extinction coefficient of 87,210 M^−1^·cm^−1^ calculated based on Pace et al*. *(Pace et al. 1995).

### Size-exclusion chromatography

The enzyme solution concentrated with Amicon Ultra 10,000 molecular weight cut-off to 0.5 mg/ml (500 μl) was loaded onto a Superdex™ 200GL column (24 ml; Cytiva) equilibrated with 50 mM Tris–HCl buffer (pH 8.0) containing 150 mM NaCl, and then the target enzyme was eluted with the same buffer. This analysis by size-exclusion chromatography was carried out using an AKTA prime plus chromatography system (Cytiva). Ovalbumin (44 kDa), conalbumin (75 kDa), aldolase (158 kDa), ferritin (440 kDa), and thyroglobulin (669 kDa) (Cytiva) were used as molecular weight markers. Blue dextran 2000 (2,000 kDa) was used to determine the void volume of the column. The molecular weight of TiCGS_Cy_ was calculated using Eq. 1.

1 $${K}_{av}=\frac{{V}_{e}-{V}_{o}}{{V}_{t}-{V}_{o}}$$

where* K*_av_ is the gel-phase distribution coefficient, *V*_e_ is the volume required to elute each protein, *V*_o_ is the volume required to elute blue dextran 2000, and *V*_t_ is the bed volume of the column.

### Analysis of the cyclization activity of TiCGS_Cy_

The enzymatic reaction of TiCGS_Cy_ on LβGs was performed in 20 mM sodium acetate buffer (pH 5.0) containing 1 mg/ml of TiCGS_Cy_ and 0.4% LβGs at 30 °C for one hour. After a heat treatment at 100 °C for 5 min, the sample (20 µl) was mixed with 20 µl of 0.1 mg/ml β-glucosidase from *Bacteroides thetaiotaomicron* (BGL) (Ishiguro et al. 2017) in 100 mM sodium acetate buffer (pH 5.0) and incubated at 40 °C for 30 min or 60 min. After a heat treatment at 100 °C for 5 min, the sample (20 µl) was mixed with 20 µl of 0.2 mg/ml CpSGL in 100 mM sodium acetate buffer (pH 5.0). The reaction mixture was incubated at 30 °C for an hour. Each reaction mixture was analyzed by thin layer chromatography (TLC).

### TLC analysis

The reaction mixtures (0.5, 1, or 2 µl) were spotted onto TLC Silica Gel 60 F_254_ plates (Merck). As for analysis of cyclization activity, the plates were resolved with 70% acetonitrile. In the case of glucose and Sop_2-5_ produced by TiCGS_Cy_, the plates were resolved with 75% acetonitrile. In the case of Sop_6-10_, the plates were resolved twice with the solution (1-butanol: acetic acid: deionized water = 2:1:1). The plates were then soaked in a 5% sulfuric acid/ethanol solution (w/v) and heated in an oven until the spots were clearly visualized.

The enzymatic reactions of TiCGS_Cy_ with each substrate (0.03% arabinogalactan, 0.2% CM-pachyman, 0.2% laminarin, 0.2% CM-cellulose, 0.2% CM-curdlan, 0.2% arabinan, 0.2% polygalacturonic acid, 0.2% LβGs or 0.2% tamarind xyloglucan, 5 mM glucose and Sop_2–10_) were carried out in 100 mM sodium acetate buffer (pH 5.0) containing 1 mg/ml of TiCGS_Cy_ at 30 °C. After a heat treatment at 100 °C for 5 min, the reaction products were detected by TLC.

### NMR analysis

To collect the cyclic products of TiCGS_Cy_ derived from LβGs, the enzymatic reaction was performed at 30 °C for 42 h in 100 mM sodium acetate buffer (pH 5.0) containing 2 mg/ml of TiCGS_Cy_ and 5% LβGs with an average DP of 121 calculated from the number average molecular weight (Motouchi et al. 2023). After a heat treatment at 100 °C for 10 min, the supernatant (4 ml) was collected after centrifugation at 4,427 × *g*. Then the solution was mixed with 250 µl of 2.5 mg/ml BGL in 100 mM sodium acetate buffer (pH 5.0) and incubated at 30 °C for 27 h, which ensures completion of the reaction; the intensity of spot showing the polysaccharides no longer changes. After a heat treatment at 100 °C for 10 min, the sample was centrifugated at 4,427 × *g*. Then the supernatant was filtrated with a 0.45-μm filter (Sartorius). The sample was fractionated by size-exclusion chromatography using a Toyopearl HW-40F column (approximately 2 L gel), as described previously (Nakajima et al. 2014), and the fractions containing the target compound were freeze-dried using a FDU-2100 (EYELA, Tokyo, Japan). The resultant powder was dissolved in D_2_O, and acetone was added as a standard for calibration of chemical shifts. The chemical shifts were recorded relative to the signal of the methyl group of the internal standard acetone (2.22 ppm). As a reference, CβGs donated by Dr. Hisamatsu (Hisamatsu et al. 1984) were also dissolved in the same solvent. ^1^H-NMR spectra were recorded using a Bruker Avance 400 spectrometer (Bruker BioSpin).

### Mass spectrometric analysis

The samples prepared for the NMR analysis were also analyzed by mass spectrometry. The positive electrospray-ionization mass spectra (ESI/MS) were recorded with samples dissolved in H_2_O containing 5 mM ammonium acetate on a X500R QTOF mass spectrometer (Sciex, Toronto, CA).

### X-ray crystallography

The enzyme solution was concentrated to 17.6 mg/ml. The initial screening of TiCGS_Cy_ crystallization was performed using MembFac HT (Hampton research, CA, USA). The crystal for data collection was obtained by incubation of the mixture of 17.6 mg/ml TiCGS_Cy_ (2 μl) and a reservoir solution (2 μl) containing 0.1 M sodium cacodylate and 1.3 M sodium acetate (pH 6.5) at 20 °C for one month. The crystal was soaked in the reservoir solution supplemented with 25% (w/v) glycerol for cryoprotection and kept at 100 K in a nitrogen-gas stream during data collection. The X-ray diffraction data was collected on a beamline (BL-5A) at Photon Factory (Tsukuba, Japan). The TiCGS_Cy_ structure was determined by molecular replacement using a predicted TiCGS_Cy_ structure by AlphaFold2 (Jumper et al. 2021) as a model structure. The molecular replacement, auto model building, and refinement were performed using the MOLREP program (Vagin and Teplyakov 2010), REFMAC5 program (Murshudov et al. 1997), and Coot program (Emsley and Cowtan 2004), respectively. A structural homology search was performed with the DALI server (Holm 2020). The secondary structure was assigned with the DSSP program (Touw et al. 2015). The multiple amino acid alignment and the structure-based amino acid alignment with the secondary structures were visualized using the ESPript 3.0 server (http://espript.ibcp.fr/ESPript/ESPript/) (Robert and Gouet 2014). The overall structures of TiCGS_Cy_, TfSGL and CpSGL were superimposed using the PDBeFold server (https://www.ebi.ac.uk/msd-srv/ssm/ssmcite.html) (Krissinel and Henrick 2004). All the structures in the figures were designed with the PyMOL program.

### Mutational analysis

The plasmids of TiCGS_Cy_ mutants (E1442Q, E1442A, and E1356A) were constructed using a PrimeSTAR mutagenesis basal kit (Takara Bio) according to the manufacturer’s instructions. PCRs were performed using appropriate primer pairs (Table S2) and the template TiCGS_Cy_ plasmid. The transformation to *E. coli*, the expression, and purification of TiCGS_Cy_ mutants were performed in the same manner as that for the wild-type TiCGS_Cy_. The enzymatic reactions were performed basically in the same manner as in the detection of cyclization activity of TiCGS_Cy_.

## Results

### Purification of cyclization domain of CGS from T. italicus (TiCGS_Cy_)

Domain configuration and biochemical function including cyclization activity of TiCGS were totally unknown. Therefore, multiple amino acid alignment was performed using CGS homologs including TiCGS and BaCGS (Fig. S2). The region of cyclization domain was expected to be residues 1005–1591 a.a. based on the minimum region that retains cyclization activity in BaCGS (Ciocchini et al. 2006; Guidolin et al. 2009). In addition, all transmembrane regions are within residues 1–1004 a.a. according to TMHMM-2.0 server (Krogh et al. 2001). Thus, the region (1005–1591 a.a.) was defined TiCGS_Cy_, and TiCGS_Cy_ fused with histidine-tag at the C-terminus was produced as a recombinant protein. The recombinant TiCGS_Cy_ (hereafter simply called TiCGS_Cy_) was purified by nickel affinity chromatography and hydrophobic chromatography, with which we obtained highly purified TiCGS_Cy_ that migrated as a single band at approximately 70 kDa in the SDS-PAGE analysis. It is consistent with a theoretical molecular mass of TiCGS_Cy_ (69.5 kDa).

### Size-exclusion chromatography analysis of TiCGS_Cy_

To investigate quaternary structure of TiCGS_Cy_, size-exclusion chromatography was performed. TiCGS_Cy_ eluted at the retention time corresponding to 60.6 kDa, which is similar to the theoretical molecular mass shown above (Fig. S3). This result indicated that TiCGS_Cy_ exists as a monomer and TiCGS does not form multimer through interactions between the TiCGS_Cy_ domains.

### Cyclization activity of TiCGS_Cy_

**Fig. 1:**
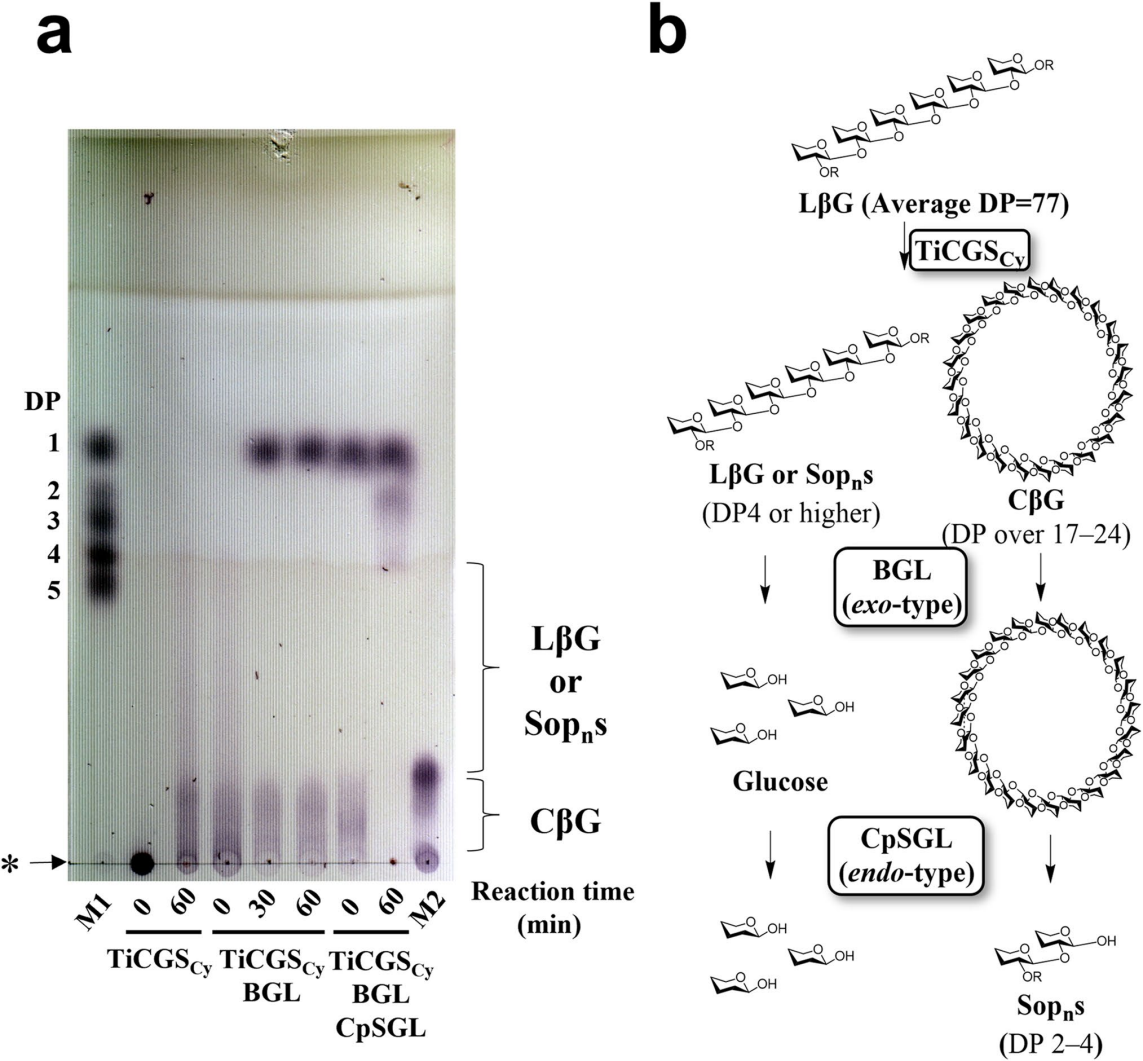
Products from catalysis of LβGs by TiCGS_Cy_. **a** Detection of the reaction products by TLC analysis. Lane M1, 5 mM glucose and Sop_2–5_. Lane M2, 0.2% CβGs with DP17–24. Each sample (0.5–2 μl) was spotted on the plate. BGL and CpSGL represent treatment of products with BGL and/or CpSGL. The asterisk represents the origin of the TLC plate.** b** The present method to distinguish between cyclic and linear forms of β-1,2-glucans. The glucose molecules are illustrated with only the β-1,2-carbon skeleton and the hydroxy group at the reducing end. 'R' represents Sop_n_s

To test the ability of purified TiCGS_Cy_ in cyclization of LβGs, the reaction products were analyzed by TLC. In the glycosylation (the former) step of the reaction by typical transglycosylases, linear glucans on the reducing end leave the catalytic site upon formation of the glycosyl-enzyme intermediate (Bissaro et al. 2015; Sinnott 1990; Van der Veen et al. 2000). In the deglycosylation (the latter) step, linear glucan products are expected to be produced in the inter-molecular transglycosylation called disproportionation. Note that the reaction with a water molecule in this step would result in hydrolysis but also releases a hydrolyzed linear product. On the other hand, cyclic products are produced when the non-reducing end of a sugar reacts with the covalent glycosyl-enzyme intermediate (reducing end) of the same molecule. If TiCGS_Cy_ possesses cyclization activity, both linear and cyclic glucan chains are expected to be produced. After incubation of LβGs with TiCGS_Cy_, a broad smear band in DPs smaller than those of the substrate was detected (Fig. 1a). Next, the reaction products were subjected to the BGL that act exolytically on the non-reducing end of LβGs (Fig. 1b). Consequently, glucose was produced, but a bit smear band with relatively higher DPs remained on the TLC plate (Fig. 1a). The BGL-treated products were further treated with CpSGL, an *endo*-type enzyme that produces Sop_2–4_ (Fig. 1b), resulting in disappearance of the smear band and appearance of Sop_2–4_ instead (Fig. 1a). These results indicate that the products that formed the smear band after the BGL treatment were in cyclic forms, and thus, TiCGS_Cy_ possesses the cyclization activity. In addition, the smear band after the BGL treatment was detected at the position lower than the spot of the marker CβGs with DP17–24 (lane M2) in the TLC plate (Fig. 1a), suggesting that DPs of the cyclic products released by TiCGS_Cy_ are higher than 17–24. Furthermore, various polysaccharides were examined as candidate substrates by TLC analysis, but no reaction was detected (Fig. S4). This result suggested that the cyclization activity of TiCGS_Cy_ is highly specific to LβGs.

### NMR analysis of compounds produced from LβGs by TiCGS_Cy_

In order to identify cyclic glucans produced by TiCGS_Cy_, the reaction product from LβGs was treated with BGL, and the BGL-resistant products were then purified by size-exclusion chromatography. The chemical shifts of the resultants measured by ^1^H-NMR were almost the same as those of the reference (Figs. S5a and b) (Hisamatsu et al. 1984; Roset et al. 2006). In addition, chemical shifts derived from H-2 and H-4 at the non-reducing end glucose moiety and H-1 (α-anomer) at the reducing end glucose moiety as in the case of LβGs (Nakajima et al. 2014) (Fig. S5c) were not detected (Fig. S5a). These facts indicate that TiCGS_Cy_ produces CβGs, and this enzyme follows an anomer-retaining mechanism.

### ESI/MS analysis of compounds produced from LβGs by TiCGS_Cy_

To investigate the DP distribution of CβGs synthesized by TiCGS_Cy_ from LβGs, the NMR products (mentioned previously) were analyzed by the positive ESI/MS. As a result, multiple peaks corresponding to doubly and triply charged ions containing two or three ammonium ions were detected (Fig. S6a). Furthermore, these peaks matched the theoretical m/z of CβGs with DP17–26 (Fig. S6b). These results suggest that TiCGS_Cy_ synthesizes CβGs with DP17–26.

### Action patterns of TiCGS_Cy_

**Fig. 2:**
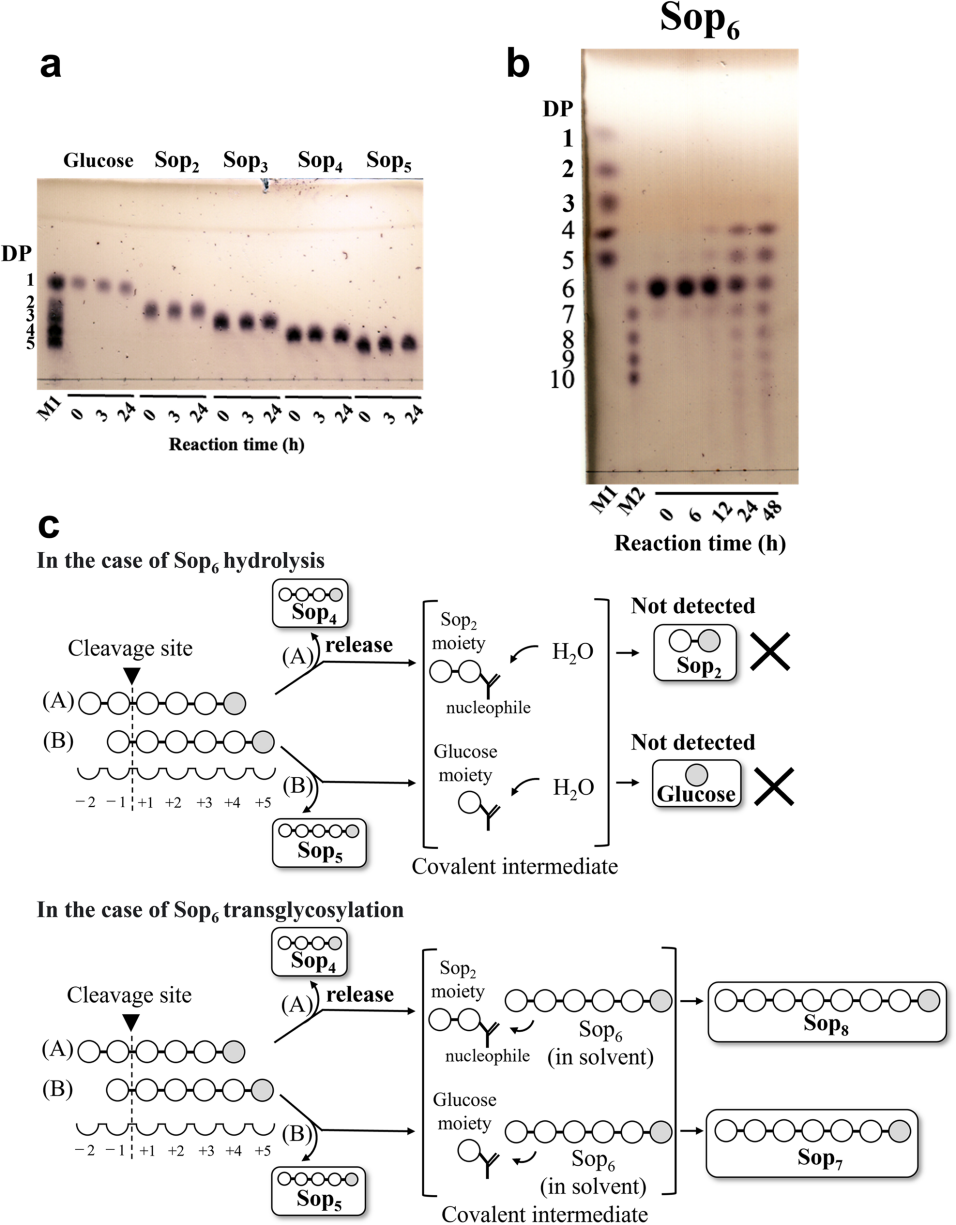
TLC analyses of activity toward glucose and Sop_2–6_. **a, b** Lane M1, 5 mM glucose and Sop_2–5_. Lane M2, 5 mM Sop_6–10_. Each sample (0.2–1 μl) was spotted on the plate. **c** Possible reaction steps of TiCGS_Cy_ with a substrate Sop_6_. Subsite binding is based on TLC results. The hypothesized upper two patterns of the hydrolytic reaction were not actually observed because H_2_O molecules do not participate in attacking covalent intermediates. The glucose molecule at the reducing end is shown in gray

**Fig. 3:**
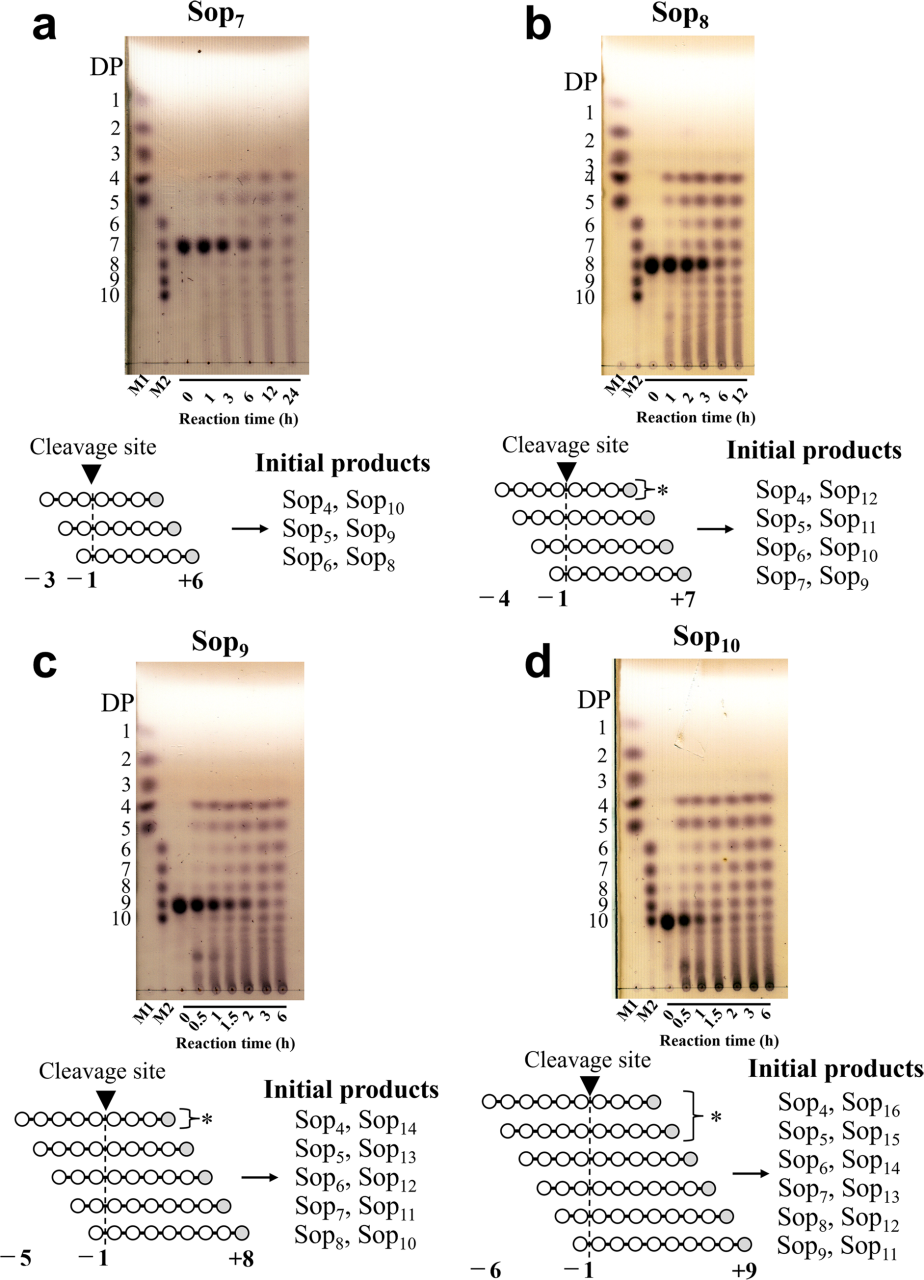
TLC analysis of activity toward Sop_7–10_. Lane M1, 5 mM glucose, and Sop_2–5_. Lane M2, 5 mM Sop_6–10_. Asterisks represent preferential reaction patterns at the initial stage of the reactions

To clarify chain length specificity of substrates, various Sop_n_s were adopted in the experiments. In the case of glucose and Sop_2–5_, no reaction product was detected by TLC analysis (Fig. 2a). On the contrary, Sop_n_s with DPs 4 or higher were produced when Sop_n_s with DPs 6 or higher were applied as substrates (Figs. 2b and 3). These results indicate that specific substrates of TiCGS_Cy_ in transglycosylation is Sop_n_s with DPs 6 or higher.

Generally, in the case of enzymes that produce cyclic glycan polymers, a nucleophilic amino acid sidechain initially binds covalently to a substrate to form a glycosyl-enzyme intermediate (Bissaro et al. 2015). This intermediate is then subjected to nucleophilic attack either by a water molecule, an intermolecular hydroxy group, or an intramolecular hydroxy group to cause hydrolysis, disproportionation or cyclization reaction, respectively (Bissaro et al. 2015; Sinnott 1990; Van der Veen et al. 2000). In the case of TiCGS_Cy_ with Sop_6_, Sop_4_ and Sop_5_ were detected as a result of reaction, which suggested that Sop_6_ binds to TiCGS_Cy_ at the catalytic site from subsite –2 to subsite + 4 or from –1 to + 5 (Figs. 2b and 2c). However, glucose and Sop_2_ (counterparts of Sop_5_ and Sop_4_, respectively, when Sop_6_ is hydrolyzed) were not detected. This result indicates that TiCGS_Cy_ catalyzes only transglycosylation without hydrolysis (Fig. 2c). Likewise, with Sop_7–10_ as substrates, glucose and Sop_2–3_ were not detected (Fig. 3). Therefore, TiCGS_Cy_ requires at least four subsites (from subsite + 1 to subsite + 4) occupied by glucose moieties for the reaction to proceed. The reaction mechanism of cyclization is fundamentally the same as that of transglycosylation. The only difference is whether the reaction is intra-molecular or inter-molecular. This is one of the reasons why linear products can be generated by CGS_Cy_. The transglycosylation reaction results in linear products when intramolecular cyclization is not accomplished due to shortage in lengths of the substrate. As the minimum DP of the synthesized CβG is 17 according to the ESI/MS results, the initial reaction products from Sop_6–10_ are not cyclic.

In terms of the reaction velocities of substrates examined, the larger the DPs of the substrates were, the faster the amounts of the products reached to the similar level (Figs. 2 and 3). This result suggests that TiCGS_Cy_ prefers longer substrates, which is consistent with the biochemical property of CGS known to produce CβGs with DPs around 20. The amount of Sop_4–6_ produced from Sop_7_, Sop_8_ and Sop_9_ at the initial stage of the reactions were Sop_4_ > Sop_5_ > Sop_6_, while in the case of Sop_10_ as a substrate, it was Sop_4–5_ > Sop_6_ (Figs. 2 and 3). These results suggest that at least from subsite − 1 to subsite − 5 in subsite minus side are involved in substrate recognition.

### Overall structure of TiCGS_Cy_

**Fig. 4:**
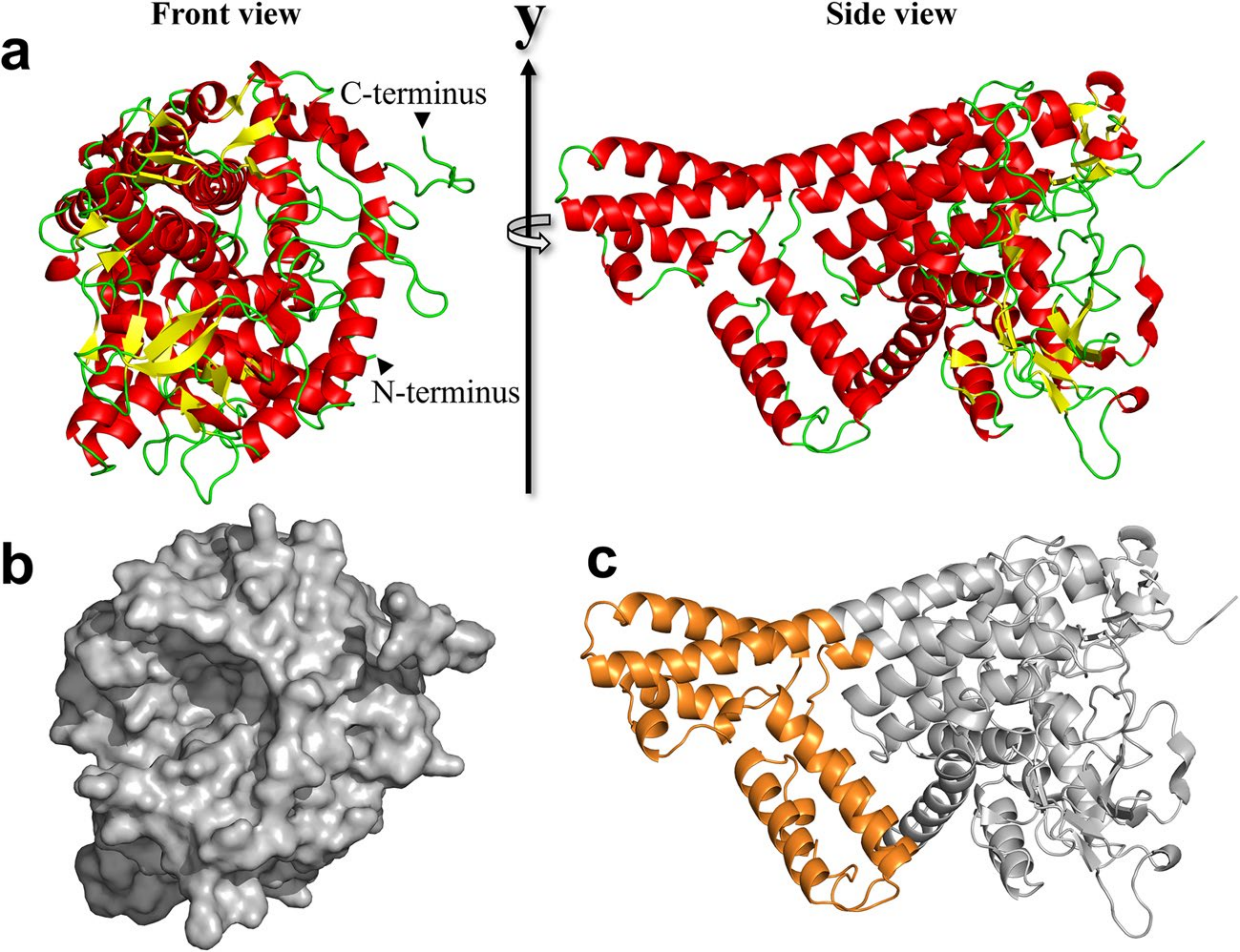
Overall structure of TiCGS_Cy_. Cartoon (**a, c**) and surface (**b**) representations of TiCGS_Cy_. α-Helices and β-strands are shown in red and yellow, respectively. The surface is shown in gray. The additional α-helices observed in TiCGS_Cy_ but not in TfSGL and CpSGL is shown in orange

A ligand-free structure of TiCGS_Cy_ was determined at 3.9 Å resolution (Table S1). An asymmetric unit contains almost identical four molecules (RMSD, 0.3 Å). The enzyme consists of a single (α/α)_6_-barrel domain with several inserted α-helices (Fig. 4a, c). According to DALI server (Holm 2020), CpSGL (RMSD, 2.4 Å; sequence identity, 17%; PDB ID, 5GZH), GH144 enzyme from *Parabacteroides distasonis* (RMSD, 2.4 Å; sequence identity, 18%; PDB ID, 5Z06), and TfSGL (RMSD, 2.7 Å; sequence identity, 12%; PDB ID, 6IMU) came up as top 3 structurally similar proteins in the case TiCGS_Cy_ is set as a query structure. TiCGS_Cy_ is structurally similar to these three enzymes although amino acid sequence identities are very low. Structure-based multiple amino acid alignment suggests that the additional α-helices in the middle region is unique to CGSs, and they are not found in SGLs (Fig. 4c). A large pocket observed in (α/α)_6_-barrel domain is expected to be a substrate binding site of TiCGS_Cy_ (Fig. 4b).

### Comparison of substrate-binding site of TiCGS_Cy_ with CpSGL and TfSGL

**Fig. 5:**
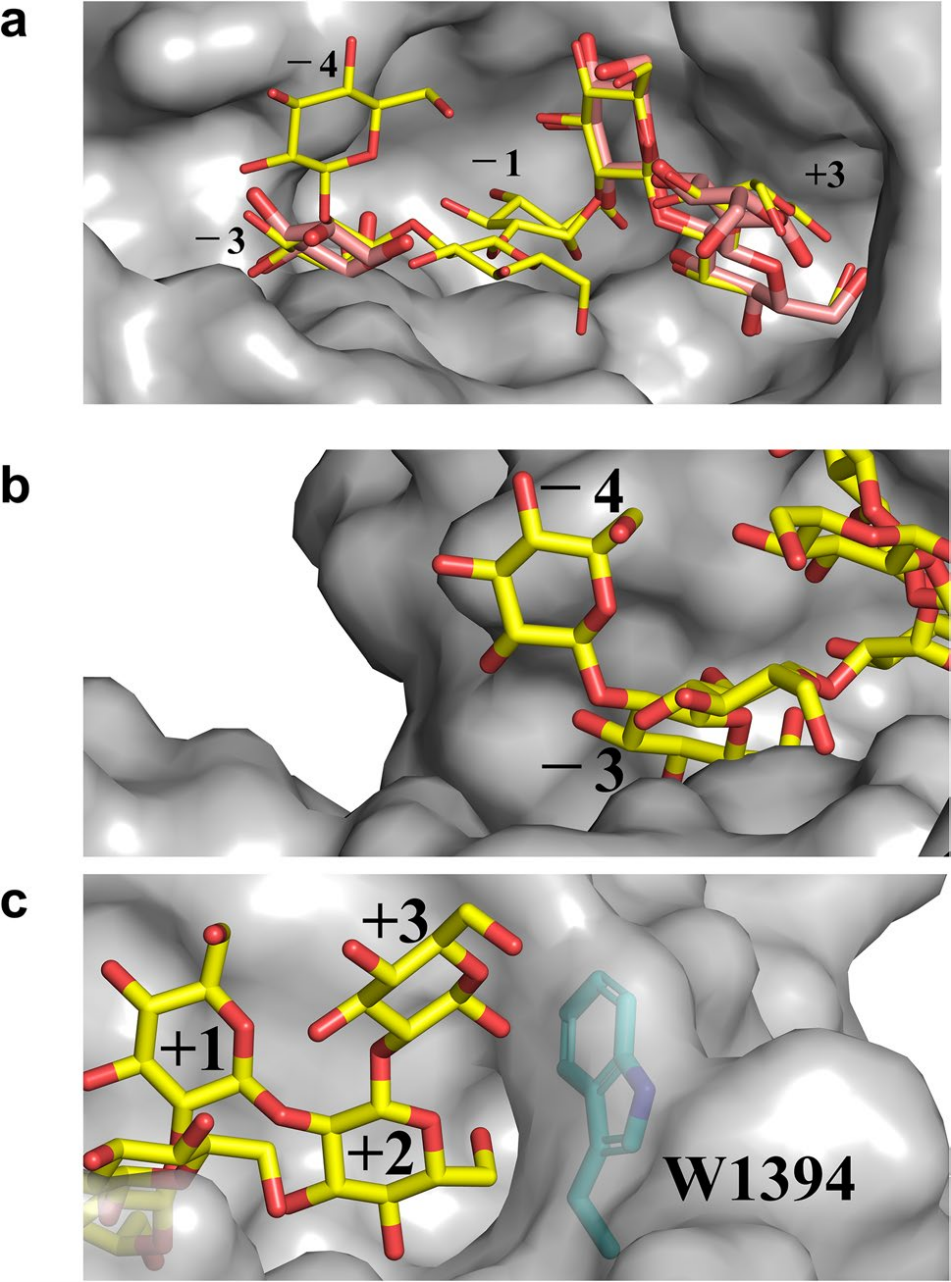
Substrate pocket of TiCGS_Cy_. The substrate pocket of TiCGS_Cy_ is shown semi-transparently in gray. Sop_7_ molecules shown as yellow sticks are placed by superimposition of TfSGL-Sop_7_ complex structure. Glucose and Sop_3_ molecules shown as light red sticks are placed by superimposition of CpSGL-glucose, Sop_3_ complex structure. Number labels represent subsite positions. **b**, **c** Close-up views around subsites − 4 and + 3. PDB IDs of TfSGL and CpSGL used throughout the manuscript are 6IMW and 5GZK, respectively

To analyze a substrate binding mode of TiCGS_Cy_, TiCGS_Cy_ was superimposed with two enzymes: TfSGL (GH162) complexed with a substrate (Sop_7_) (PDB ID: 6IMW) and CpSGL (GH144) complexed with a glucose and a Sop_3_ (PDB ID: 5GZK) (Tanaka et al. 2019; Abe et al. 2017). Consequently, the three overall structures are superimposed well (Fig. S7). The shape of the substrate pocket of TiCGS_Cy_ is similar to those of TfSGL and CpSGL in that the substrates observed in TfSGL and CpSGL complex structures can be potentially accommodated in the pocket, although the superimposed glucose moiety at subsite − 3 is a little too close to TiCGS_Cy_ (Fig. 5a). There is a sufficient space beyond 2-hydroxy group of the potential subsite − 4 (Fig. 5b), which is consistent with the fact that TiCGS_Cy_ prefers Sop_n_s with larger DPs (Figs. 2 and 3). Contrarily, W1394 is likely to block binding of glucose moieties beyond subsite + 3 (Fig. 5c). Taking into account the result of action pattern analysis that subsite + 4 should be occupied (Figs. 2 and 3), side chain of W1394 may flip to make room for substrate binding. In addition, less information was retrieved by a sequence alignment among TiCGS_Cy_ homologs. Therefore, we further conducted structural alignment of TiCGS_Cy_, TfSGL and CpSGL. As a result, several residues (G1104, W1109, L1406, Y1456, V1475, P1478 and G1509) are identified as conserved residues. Among these, W1109 and Y1456, which are located near subsite − 3, are assumed to be the key residues for substrate recognition (Figs. S2 and 7). Overall, it is suggested that the pocket in the (α/α)_6_-barrel domain is the substrate binding site.

### Catalytic residues of TiCGS_Cy_

Canonical enzymes that synthesize cyclic carbohydrates take advantage of anomer-retaining mechanism to achieve transglycosylation reaction (see https://www.cazypedia.org/index.php/Transglycosylases for details) (The CAZypedia Consortium 2018; Sinnott 1990). First, an acidic residue (a nucleophile) attacks an anomeric carbon atom at subsite − 1 to form a glycosyl-enzyme covalently bonded intermediate, and an acid/base catalyst provides a proton to a scissile bond oxygen atom in a substrate to release a moiety at the reducing end from the scissile bond. This step is called glycosylation step. In the next step called deglycosylation, the intermediate is attacked by an intramolecular hydroxy group to complete a cyclization reaction mediated by an acid/base catalyst.

**Fig. 6:**
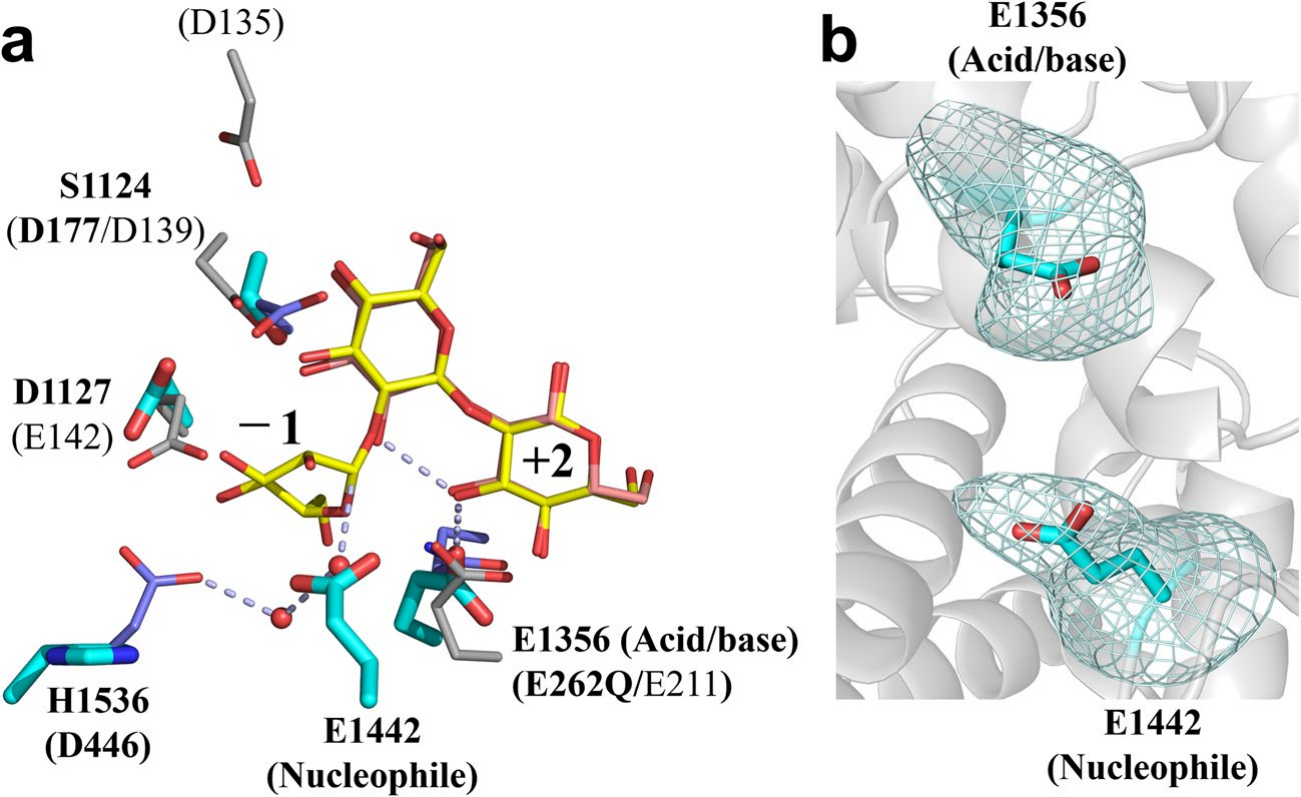
Superimposition of catalytic residues and related residues in TiCGS_Cy_, TfSGL, and CpSGL. **a** Sop_7_ in TfSGL-Sop_7_ complex and Sop_3_ in CpSGL in CpSGL-Glc, and Sop_3_ complex are partially visualized as yellow and light red sticks, respectively. Residues in TiCGS_Cy_, TfSGL, and CpSGL are shown as thick cyan, purple and gray sticks, respectively. Residues in TiCGS_Cy_, TfSGL, and CpSGL are labelled with bold letters, bold letters in parentheses and plane letters in parentheses, respectively. Water molecules observed in TfSGL-Sop_7_ complex are shown as red spheres. Gray dashed lines represent a route of the reaction pathway in TfSGL. Subsite positions are labelled − 1 and + 2. **b** A *F*_o_-*F*_c_ omit map for E1356 and E1442 in TiCGS_Cy_. The map is shown at the 4.0σ contour level and represented as cyan meshes

In the case of TiCGS_Cy_, E1442 is found with a clear electron density at the position corresponding to that of the nucleophilic water in TfSGL, which attacks the anomeric carbon of the glucose moiety at subsite − 1 (Fig. 6). Meanwhile, no candidate acidic residue directly interacting with a scissile bond oxygen atom is found. However, E1356 of TiCGS_Cy_ is well-superimposed with E262 of TfSGL, a clearly evidenced catalytic residue acting on a scissile bond of a substrate through 3-OH of a glucose moiety at subsite + 2 (Fig. 6a). Electron density of E1356 was also observed clearly (Fig. 6b). Both E1442Q and E1442A mutants showed no cyclization activity toward LβGs. In addition, the BGL-resistant products remained as a weak spot at the origin on the TLC in the case of E1356A, indicating that E1356A mutant showed very low cyclization activity toward LβGs (Fig. S8). These results strongly suggest that E1442 is a catalytic residue that acts as a nucleophile, and E1356 is an acid/base catalyst.

According to prediction of p*K*_a_ by PROPKA3.5.0 (Olsson et al. 2011), p*K*_a_ values of E1442 and E1356 in chain A were 6.72 and 8.98, respectively. E1400 highly conserved among CGSs is found in the vicinity of E1356 although E1400 is not conserved in TfSGL or CpSGL (Figs. S2 and 7). A negative charge of E1400 is considered to raise the p*K*_a_ value of E1356. Contrarily, the basic residues H1536 and H1537 are found in close proximity to E1442, which probably contribute to the decrease in p*K*_a_ value of E1442. In addition, these two histidine residues are also highly conserved among CGSs (Fig. S2). The difference of the p*K*_a_ values between E1356 and E1442 suggests that these two residues are catalysts.

**Fig. 7:**
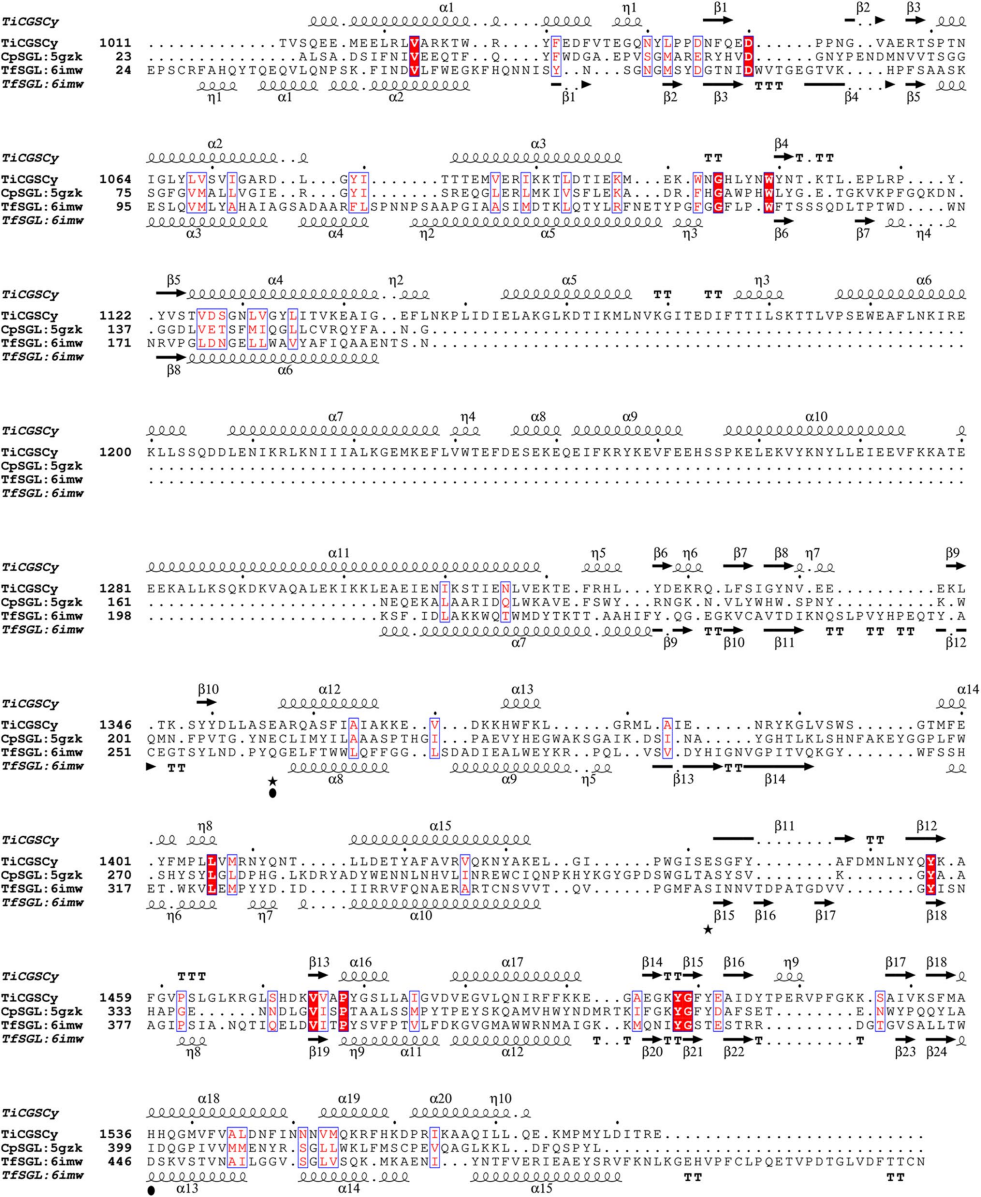
Structure-based amino acid alignment of TiCGS_Cy_, TfSGL, and CpSGL. Closed stars and circles represent catalytic residues of TiCGS_Cy_ and TfSGL, respectively

## Discussion

In the present study, we explicitly showed that TiCGS_Cy_ domain alone produced CβGs by transglycosylation reaction without hydrolysis. Whether the final reaction product is cyclic or linear solely depends on the chain length of the substrate. ESI/MS analysis revealed that the minimum DP of BGL-resistant compounds (i.e., CβGs) synthesized from substrate LβGs by TiCGS_Cy_ was 17 (Fig. S6). On the other hand, action patterns in the TLC analysis of TiCGS_Cy_ (Figs. 2 and 3) suggested that a minimum DP of 4 is additionally required at the reducing end of the cleavage site. Taken together, the minimum DP of the substrate in the synthesis of cyclic sugars is 21 (= 17 + 4). Preference of this domain for Sop_n_s with higher DPs is consistent with the chain lengths of reaction products by the intact CGSs (Hisamatsu 1992; Ciocchini et al. 2007; Guidolin et al. 2009).

Recently, a cryo-EM structure of an intact CGS from *A. tumefaciens* has been reported (Sedzicki et al. 2023). Nevertheless, a detailed reaction mechanism could not be determined due to the following reasons: Reaction products from LβGs have not been identified as CβGs; With the whole CGS, it was difficult to distinguish between activities of different domains, and the possibility that GH94 glycoside phosphorylase domain produced LβGs by transglycosylation in reversible reactions of phosphorolysis could not be excluded; A plausible reaction pathway to account for transglycosylation could not be drawn from the structure because the substrate chain appears to be placed in reverse orientation in comparison with that determined in TfSGL.

**Fig. 8:**
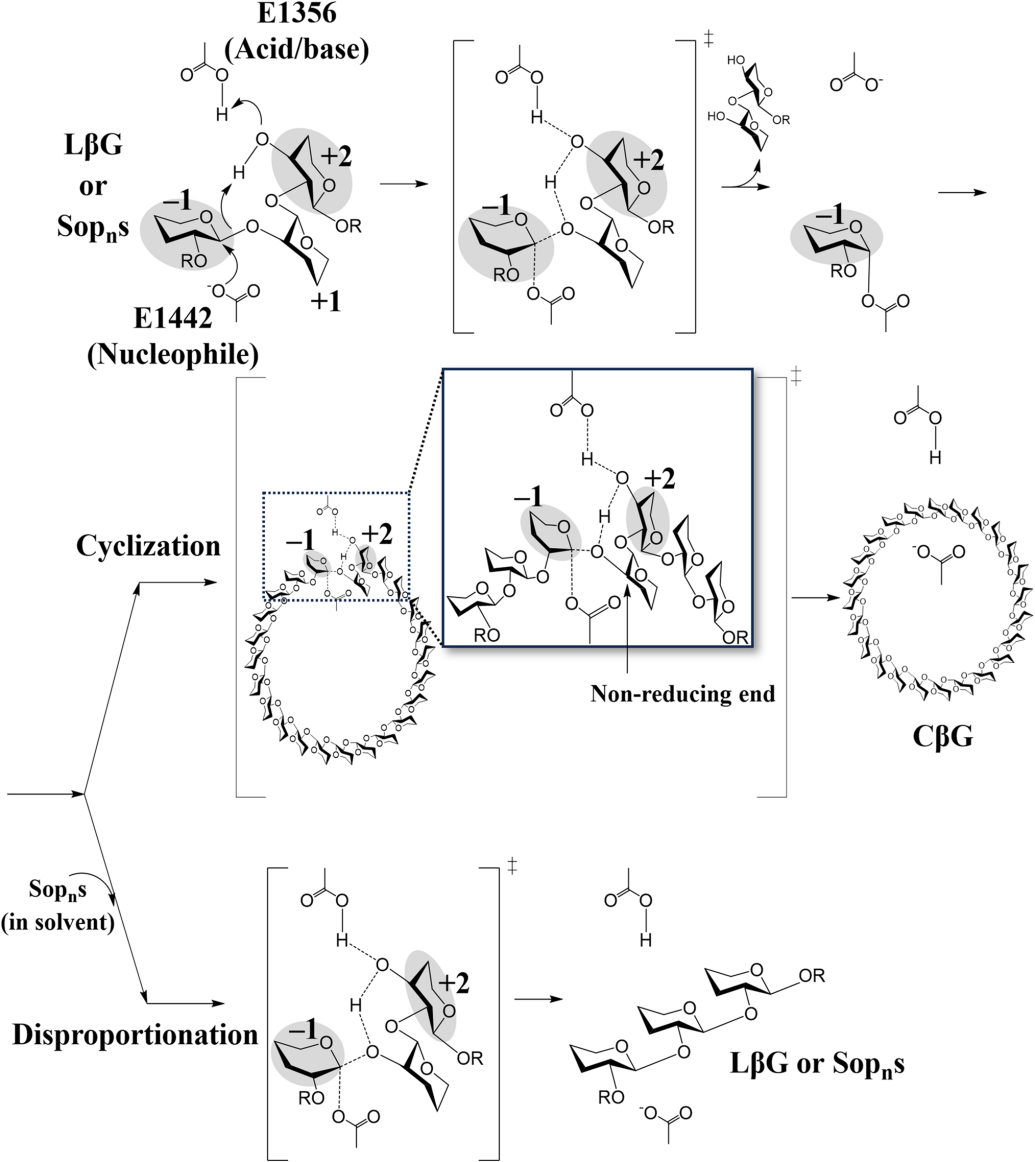
Schematic representation of the proposed reaction mechanism of TiCGS_Cy_. The glucose molecules are illustrated with only the β-1,2-carbon skeleton. Only the hydroxy group at the reducing end and the 3-hydroxy group of the glucose molecule at subsite+2 are shown. 'R' represents Sop_n_s

With clarified enzymatic functions and a solid reaction pathway of the sole TiCGS_Cy_, the present study is the first demonstration of detailed reaction mechanism of the CGS_Cy_ domain. Based on the overall results of structural and functional analysis of TiCGS_Cy_, the reaction pathway of the enzyme can be explained as follows (Figs. 6 and 8). First, in the glycosylation step, E1356 acts as a general acid to provide a proton to a scissile bond oxygen atom through 3-OH group of a glucose moiety at subsite + 2. Simultaneously, E1442 attacks an anomeric center at subsite − 1 as a nucleophile to form a glycosyl-enzyme intermediate. Next, E1356 acts as a general base to draw a proton of inter- or intra-molecular 2-OH group of a glucose moiety at subsite + 1 through 3-OH group of a glucose moiety at subsite + 2. The activated (deprotonated) 2-OH group at subsite + 1 attacks the anomeric carbon of covalently bonded glucose moiety at subsite − 1 to release transglycosylation products. If an intermolecular hydroxy group, which belongs to a different molecule, attacks an anomeric carbon atom, a product in a linear form is released. In the case of an intramolecular hydroxy group, a product in a cyclic form is released (Fig. 8).

Considering the unique reaction mechanism of TiCGS_Cy_ involving the 3-OH group of the glucosyl residue at the subsite + 2, it is evident that the well-ordered pre-association of the oligosaccharide acceptor is essential for TiCGS_Cy_ to perform effective transglycosylation. This process is unlikely to be replaced by individual water molecules, as they do not form such arrangements efficiently or frequently due to entropic factors. This might be the reason why TiCGS_Cy_ does not perform hydrolysis, and why the glycosyl-enzyme remains stable until oligosaccharide donors enter and bind to the positive subsites appropriately.

Furthermore, although such proton transfer called Grotthuss mechanism is non-canonical among GH families (de Grotthuss 1806; Cukierman 2006), GH162 and GH186 SGLs (TfSGL and OpgD from *E. coli*, respectively) (Tanaka et al. 2019; Motouchi et al. 2023), GH130 4-*O*-β-d-mannosyl-d-glucose phosphorylase (Nakae et al. 2013), and GH136 lacto-*N*-biosidase (Yamada et al. 2017) share this exceptional Grotthuss mechanism in GH families, supporting the proposed reaction mechanism of TiCGS_Cy_.

Comparison of the reaction mechanisms between GH144, GH162, and CGS revealed that the general acid (E262 in GH162 TfSGL) is found also in GH144 CpSGL (E211) and TiCGS_Cy_ (E1356, in anomer-retaining mechanism, an acid/base) (Fig. 6a). These residues are also conserved according to multiple amino acid alignment (Fig. 7). Contrarily, D446 in TfSGL (a general base) is substituted with a hydrophobic residue in CpSGL that cannot act as a catalyst (Fig. 6a), indicating that GH162 and GH144 have distinct pathways although we should note that the reaction pathway of GH144 has not yet been fully determined (Tanaka et al. 2019). In TiCGS_Cy_, D446 of TfSGL is substituted with H1536 (Fig. 6a). This histidine is also a proton dissociative residue highly conserved among CGSs (Fig. S2). Nevertheless, E1442 is the nucleophile and H1536 is rather considered to play an important role in supporting deprotonation of E1442. This observation clearly indicates the difference in the reaction pathways between GH162 and CGSs. Although GH162 TfSGL, GH144 CpSGL, and TiCGS_Cy_ share a similar overall fold, they belong to phylogenetically far different groups. Considering the clear differences in the reaction mechanism between the three groups, the group of CGSs including TiCGS define a new GH family, GH189.

As described above, superimposition of the present TiCGS_Cy_ structure with those of GH144 CpSGL and GH162 TfSGL revealed that arrangements of general acid or acid/base residues are common in the (α/α)_6_ motif (Figs. S9 and S10). Nevertheless, they are distinctively different from other GH clans (clan GH-G, L, M, O, P, and Q) with (α/α)_6_-folds. While the general acid or acid/base catalytic residues in CpSGL (GH144), TfSGL (GH162) and TiCGS_Cy_ (GH189) are located at almost the same position, they were never superimposed well with any of the counterparts in already-existing six GH clans (Fig. S10). This finding indicated that GH144, GH162, and GH189 are closely related to each other based on the arrangement of this key residue. Nevertheless, among them, the new enzyme TiCGS_Cy_ (GH189) has an anomer-retaining mechanism unlike the other two anomer-inverting enzymes. Because GH clans are defined basically according to both similarity in structures and reaction mechanisms, the members of groups that establish a new GH clan (clan GH-S) are GH144 and GH162. Meanwhile, GH189 is so far the only family related to clan GH-S. It would rather become a member of a potential GH clan when another new GH family of a retaining mechanism with a similar arrangement of a catalytic residue is found in the future.

The present study provides significant insights into biosynthesis of the physiologically important CβGs by further understanding of structures and functions of CGSs. Moreover, this finding is a large achievement to expand the field of carbohydrate-active enzymes by adding a new group of enzymes.

## Supplementary Information

Below is the link to the electronic supplementary material.

Supplementary file1 (PDF 3.05 MB)

## Data Availability

The raw data for the TLC, size-exclusion chromatography of TiCGS_Cy_, and the ^1^H-NMR and ESI/MS (referenced in Figs. 1–3, S3-S6 and S8) are available from the corresponding authors (email: n_tanaka@rs.tus.ac.jp; m-nakajima@rs.tus.ac.jp; tmasaike@rs.tus.ac.jp) upon reasonable request. The primer details provided in Table S2 can also be obtained through the same process. The collection and refinement statistics of the Xray crystallography presented in Table S1 are accessible from the Protein Data Bank (PDB, https://www.rcsb.org/). The data of structures in Fig. 4–7, including PDB ID 8WY1 for TiCGS_Cy_, are also available from PDB.
