# Supplementary material for: Functional and structural analysis of a cyclization domain in a cyclic β-1,2-glucan synthase
Source: Appl Microbiol Biotechnol. 2024 Feb 1;108:187. doi: 10.1007/s00253-024-13013-9 (PMC10834661; doi:10.1007/s00253-024-13013-9)
Supplement: Supplementary file 1 [file 253_2024_13013_MOESM1_ESM.pdf]

Supplementary information

Applied Microbiology and Biotechnology

Functional and structural analysis of a cyclization domain in a cyclic β-1,2-glucan synthase

Nobukiyo Tanaka[1*], Ryotaro Saito[1], Kaito Kobayashi[2], Hiroyuki Nakai[3], Shogo Kamo[1], Kouji Kuramochi[1], Hayao Taguchi[1], Masahiro Nakajima[1*] and Tomoko Masaike[1*]

[1]Department of Applied Biological Science, Faculty of Science and Technology, Tokyo University of Science, 2641 Yamazaki, Noda, Chiba 278-8510, Japan,

[2]Artificial Intelligence Research Center, National Institute of Advanced Industrial Science and Technology (AIST), 2-4-7 Aomi, Koto-ku, Tokyo, 135-0064, Japan,

[3]Faculty of Agriculture, Niigata University, Niigata 950-2181, Japan.

*Corresponding author. E-mail: n_tanaka@rs.tus.ac.jp; m-nakajima@rs.tus.ac.jp; tmasaike@rs.tus.ac.jp; Tel: +81-471-24-1501; Fax: +81-471-23-9767.

Table S1 Data collection and refinement statistics

Data set	$TiCGS_{Cy}$
Beamline	BL-5A
Space group	$P4_12_12$
**Data collection**	
Unit cell parameters (Å)	a = 172.72, b = 172.72, c = 395.60
Resolution (Å)[a]	89.60–3.90 (4.01–3.90)
Total reflections[a]	1,385,923 (116,994)
Unique reflections[a]	55,512 (4,497)
Completeness (%)[a]	100.0 (99.9)
Redundancy[a]	25.0 (26.0)
Mean $I/\sigma(I)$[a]	9.8 (2.9)
R_{merge}[a]	0.47 (1.79)
R_{pim}[a]	0.13 (0.50)
$CC_{1/2}$[a]	0.99 (0.85)
**Refinement**	
Resolution (Å)	89.60–3.90
No. of unique reflections	52,696
Completeness (%)[a]	99.96
R_{work}/R_{free} (%)	19.8/23.2
**PDB entry**	8WY1

[a] Values in parentheses represent the highest resolution shell.

Table S2 Primers used in this study

Preparation of $TiCGS_{Cy}$ mutants (5'-oligonucleotide-3')		
**Mutants**	**Forward**	**Reverse**
E1442A	ATATCAGCGTCGGGATTTTACGCTTTT	TCCCGACGCTGATATGCCCCACGGAAT
E1442Q	ATATCACAGTCGGGATTTTACGCTTTT	TCCCGACTGTGATATGCCCCACGGAAT
E1356A	GCTTCAGCAGCAAGGCAAGCGAGTTTT	CCTTGCTGCTGAAGCCAACAAGTCATA

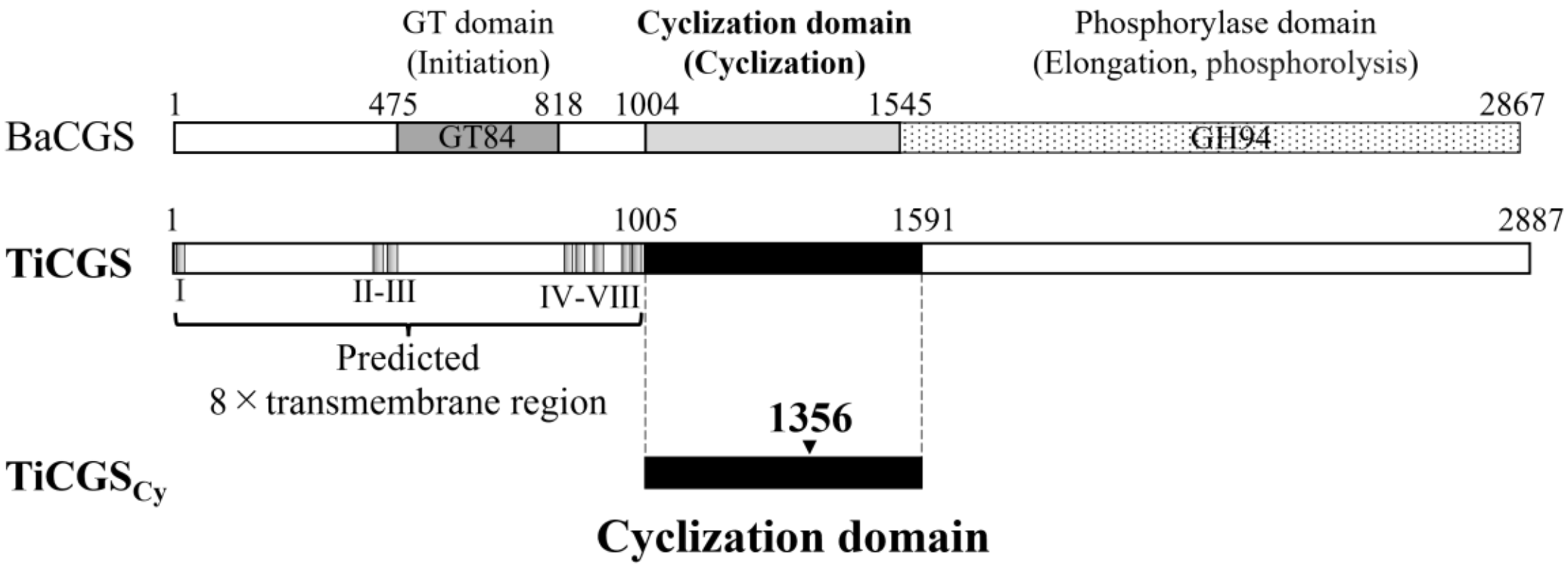

Fig. S1

The overall domain organization of BaCGSs. The GT84 domain (475–818 a.a.) of BaCGS, the cyclization domain (1004–1545 a.a.) and the GH94 domain (1545–2867 a.a.) reported in previous studies are indicated in dark gray, light gray, and dots, respectively (Guidolin et al. 2009). I to Ⅷ indicate the eight transmembrane regions (4–21, 425–447, 457–479, 832–849, 854–873, 893–915, 959–976 and 981–1004 a.a., respectively) of $TiCGS_{Cy}$ predicted by the TMHMM-2.0 server. $TiCGS_{Cy}$ (the region cloned in this article, 1005–1591 a.a.) is shown in black. By the PSI Blast search, the residue (E1356) was found as the candidate general acid conserved also in TfSGL (E262, GH162) (indicated by a black arrow).

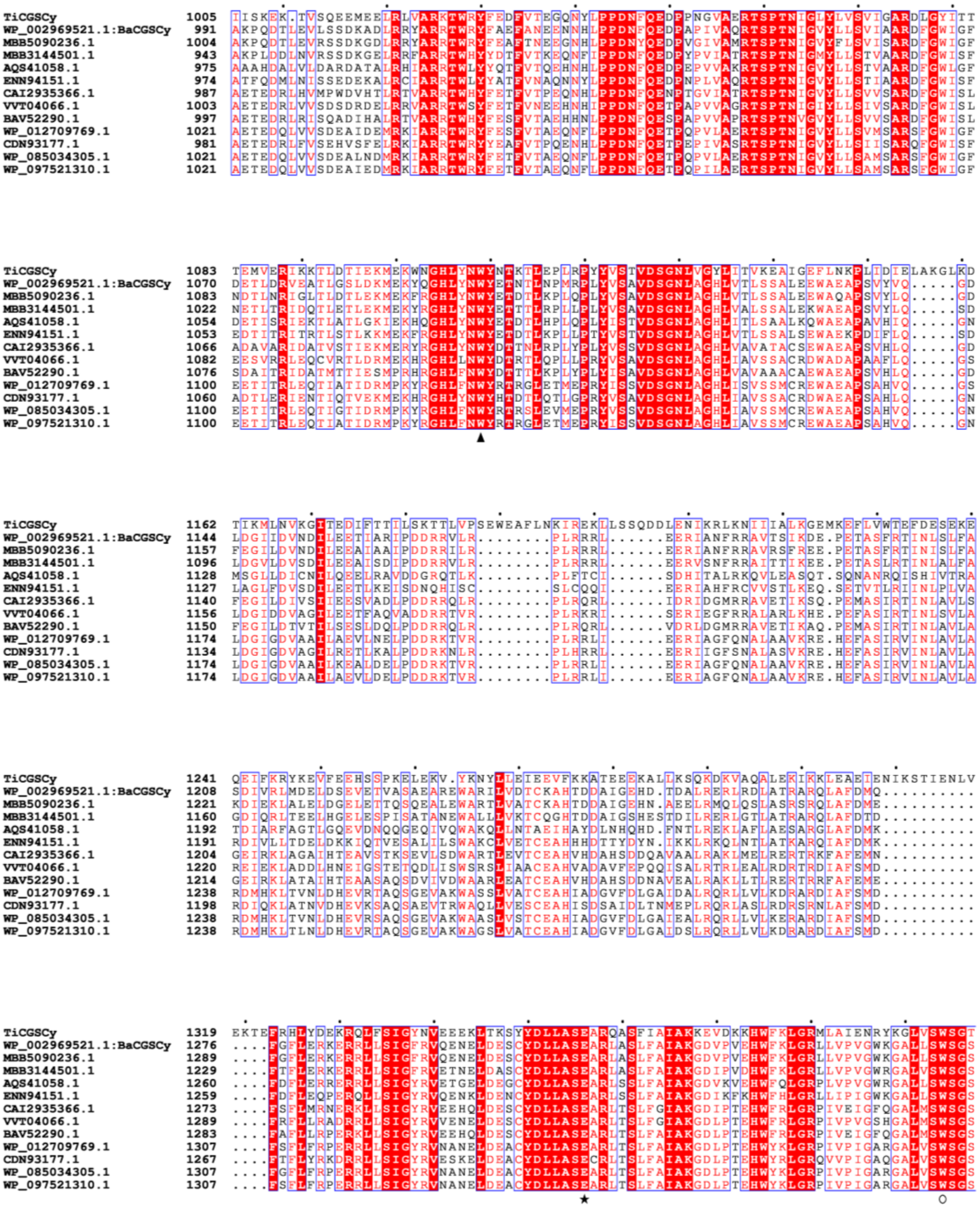

continued

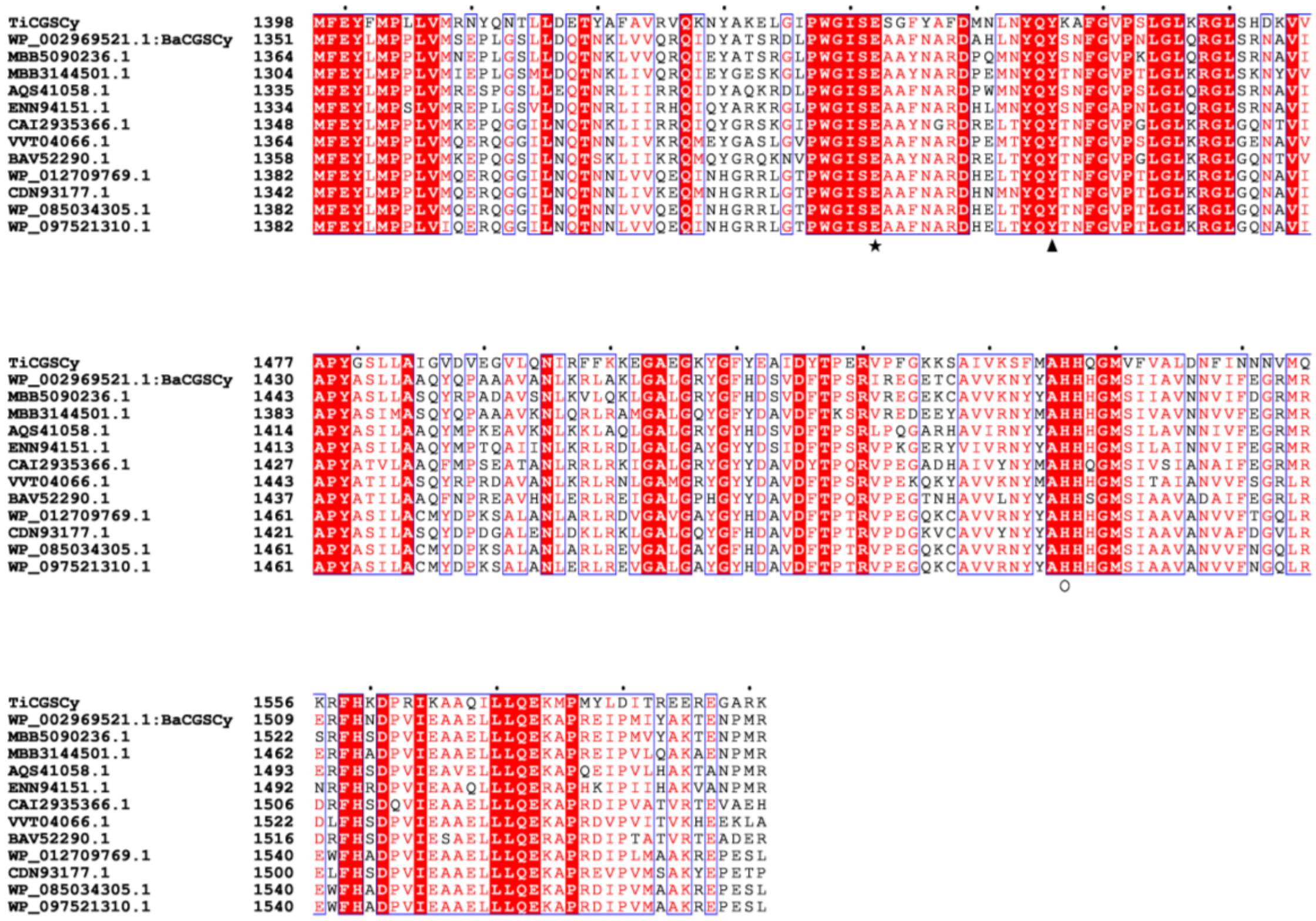

Fig. S2

Multiple amino acid alignment of CGSs. The cyclization domain region is shown. Closed stars indicate catalytic residues of TiCGS$_{Cy}$. W1394, likely blocking the binding of glucose moieties beyond the subsite +3, and H1536, a residue structurally conserved significantly with D446 of TfSGL, are labelled with open circles. W1109 and Y1456, well conserved across the homologs of TiCGS$_{Cy}$, TfSGL and CpSGL, are labelled with a black arrowhead. These residues are located at similar positions in the case of TfSGL and CpSGL.

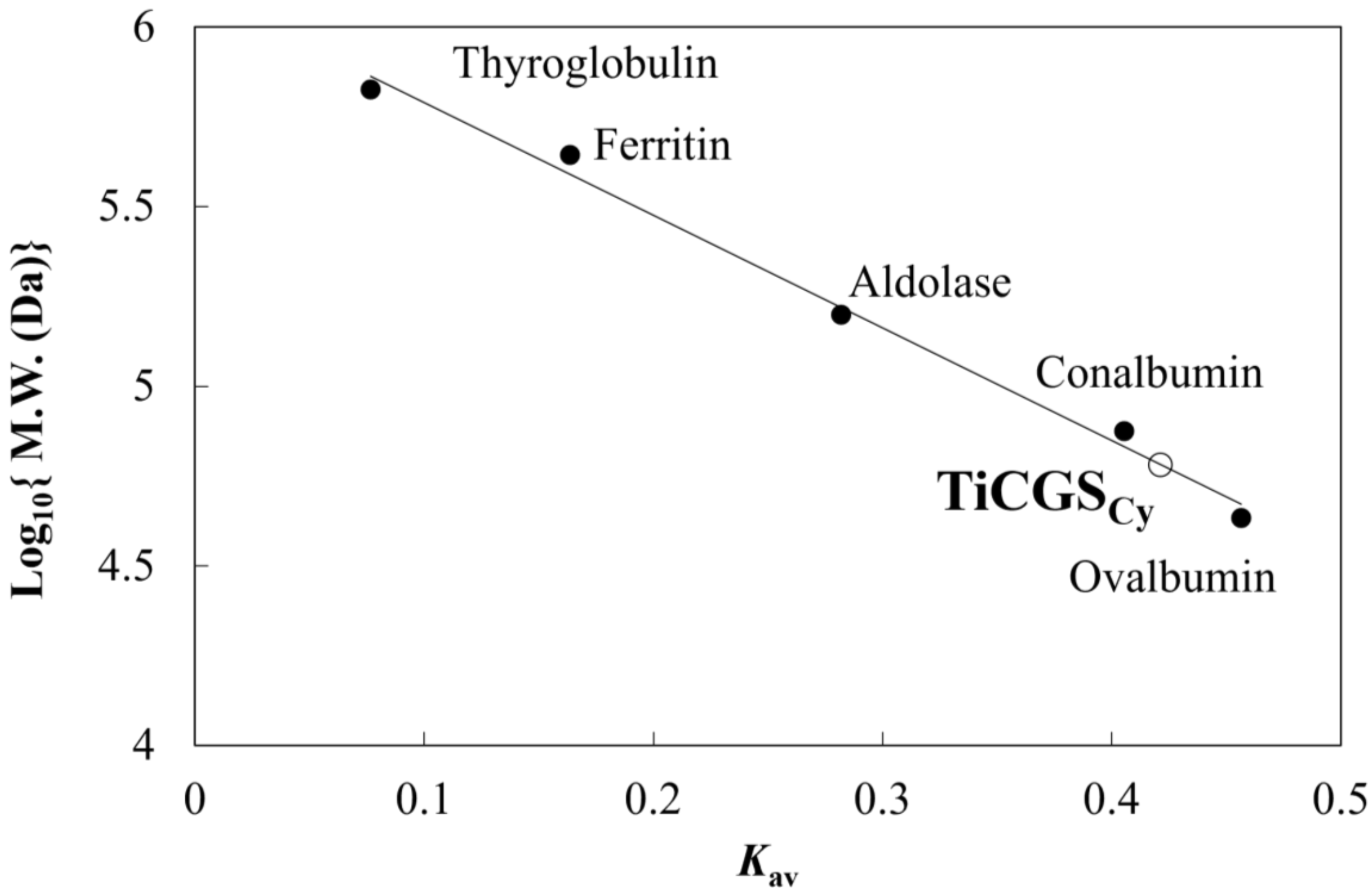

Fig. S3

Size-exclusion chromatography of $TiCGS_{Cy}$. Molecular weight protein markers and $TiCGS_{Cy}$ are shown in closed and open circles, respectively.

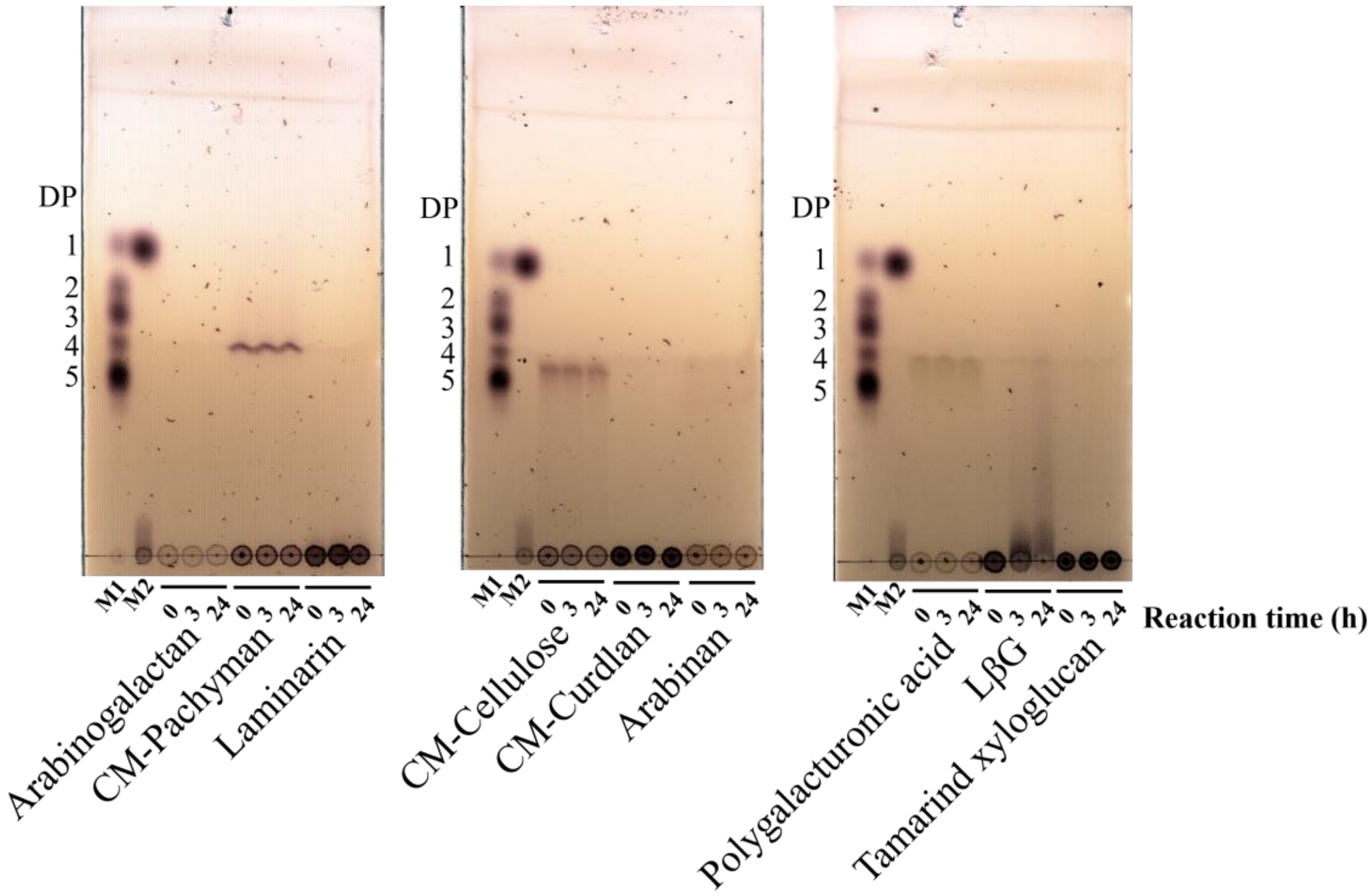

Fig. S4

Substrate specificity of TiCGS$_{Cy}$. Lane M1, 0.5 μl of mixture of glucose (5 mM) and Sop$_{2–5}$ (each 5 mM) was spotted. Lane M2, a sample for NMR after sufficient BGL-treatment. Numbers beside the TLC plates represent DPs of Glc and Sop$_{2–5}$.

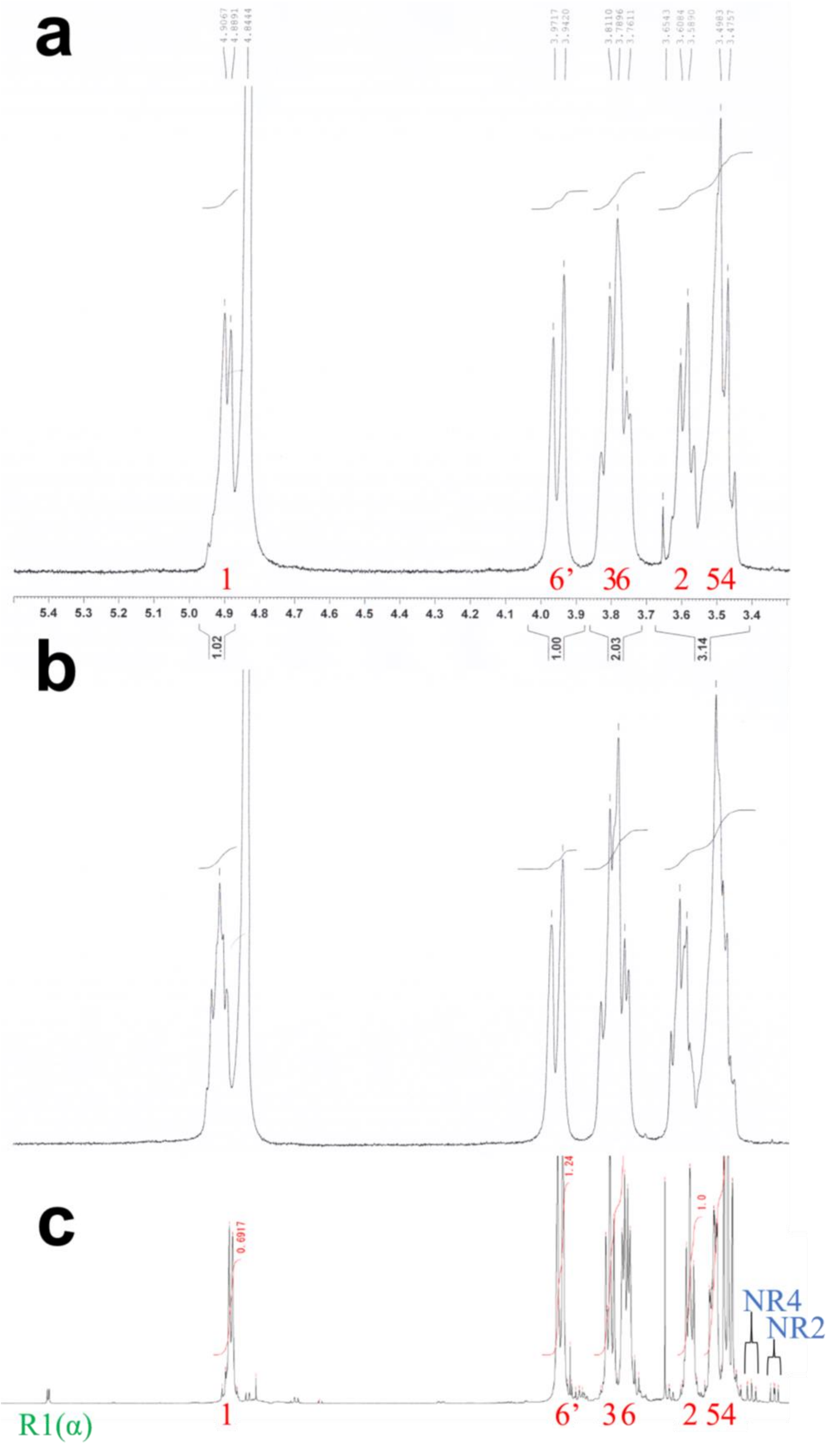

a
4.9067
4.8891
4.8444
3.9717
3.9420
3.8110
3.7896
3.7611
3.6543
3.6084
3.5890
3.4983
3.4757
1
6'
36
2
54
5.4
5.3
5.2
5.1
5.0
4.9
4.8
4.7
4.6
4.5
4.4
4.3
4.2
4.1
4.0
3.9
3.8
3.7
3.6
3.5
3.4
1.02
1.00
2.03
3.14
b
c
0.6917
1.24
1.0
NR4
NR2
R1(α)
1
6'
36
2
54

Fig. S5

^{1}H-NMR of the BGL-resistant reaction products. **a**, The BGL-resistant reaction products from LβGs catalyzed by $TiCGS_{Cy}$. **b**, CβGs with DP17–24 provided by Dr. Hisamatsu (Hisamatsu et al. 1984). **c**, LβGs with the average DP of 39 produced by SOGP from *L. innocua* (Nakajima et al. 2014). Colored numbers represent positions of protons covalently bonded to carbon atoms. Red, blue and green letters represent inner, non-reducing-end and reducing-end glucose moieties, respectively. (α) represents α-anomer glucans.

b

DP	[M + 2NH$_4$]$^{2+}$ Theoretical value (m/z)	[M + 2NH$_4$]$^{2+}$ Found peak (m/z)	**[M + 3NH$_4$]$^{3+}$ Theoretical value (m/z)**	**[M + 3NH$_4$]$^{3+}$ Found peak (m/z)**
17	**1395.5**	**1395.5**	-	-
18	**1476.5**	**1476.5**	-	-
19	**1557.5**	**1557.5**	1044.4	1044.4
20	**1638.6**	**1638.6**	1098.4	1098.4
21	**1719.6**	**1719.6**	1152.4	1152.4
22	**1800.6**	**1800.6**	1206.4	1206.4
23	**1881.6**	**1881.6**	1260.4	1260.4
24	**1962.7**	**1962.7**	1314.5	1314.5
25	-	-	1368.5	1368.5
26	-	-	1422.5	1422.5

a

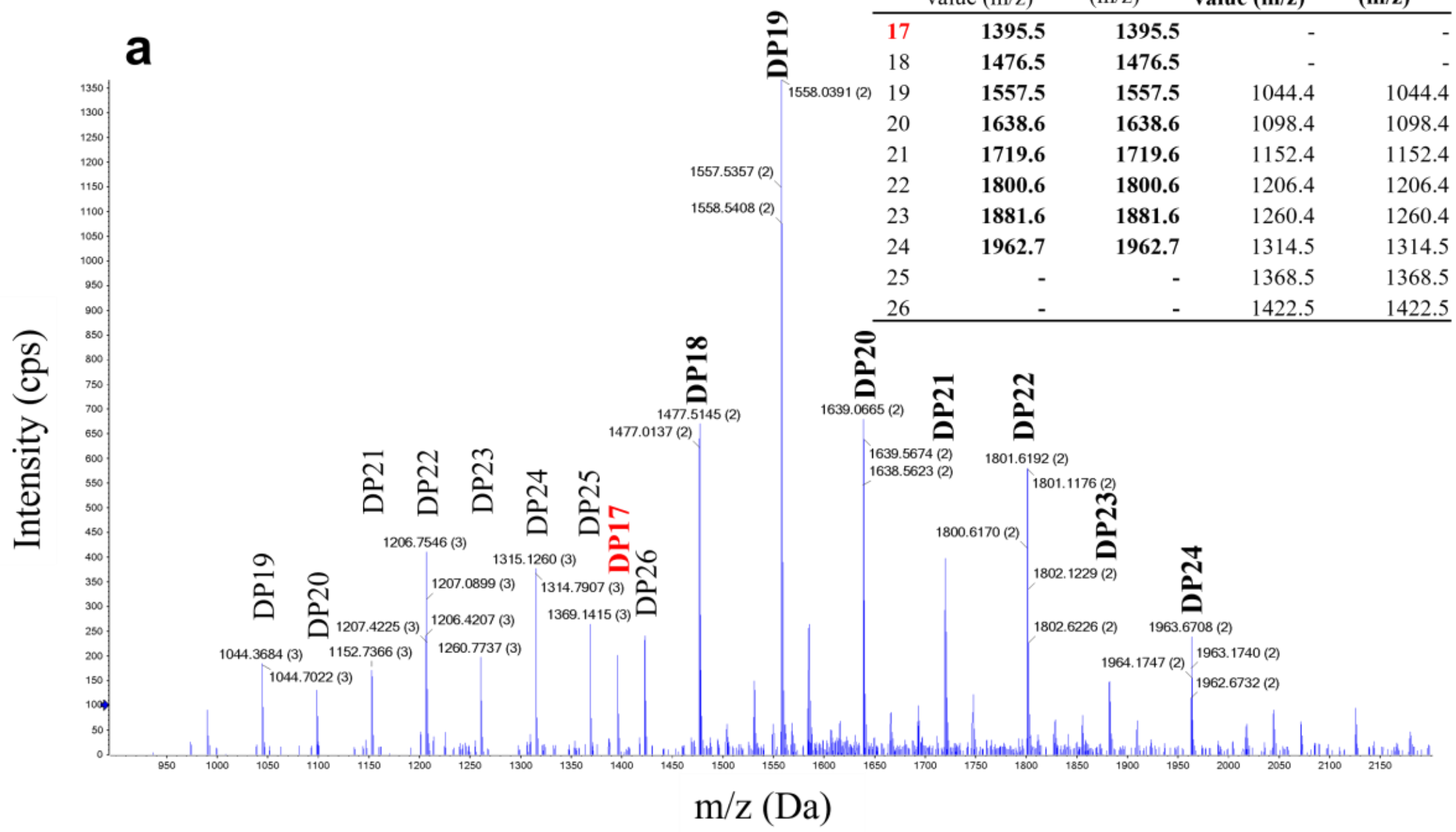

Fig. S6

Multiple ion peaks derived from CβGs in BGL-resistant products detected by positive ESI/MS analysis. **a**, The DPs of CβG in the peaks containing two or three ammonium ions are indicated in bold and plain text, respectively. The red text indicates the peak of the CβG with the minimum DP (= 17). **b**, The theoretical m/z and the detected peak m/z of CβGs with DP17–26.

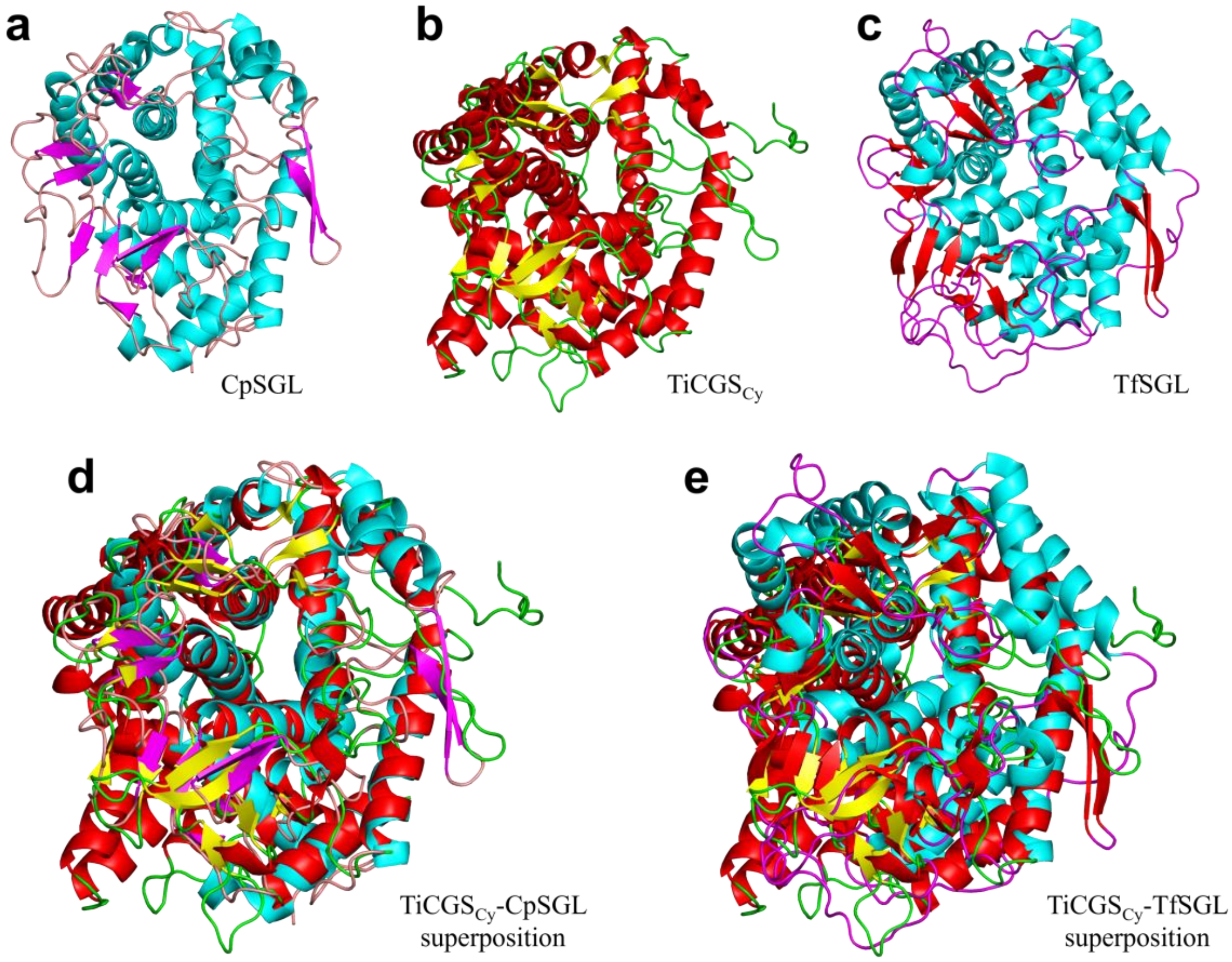

Fig. S7

Superimposition of $TiCGS_{Cy}$ with CpSGL and TfSGL. The three enzymes are shown in cartoon and are colored by secondary structures. **a**, **b**, **c**, Sole structures of CpSGL, $TiCGS_{Cy}$ and TfSGL, respectively. **d**, Superimposition of $TiCGS_{Cy}$ with CpSGL. **e**, Superimposition of $TiCGS_{Cy}$ with TfSGL.

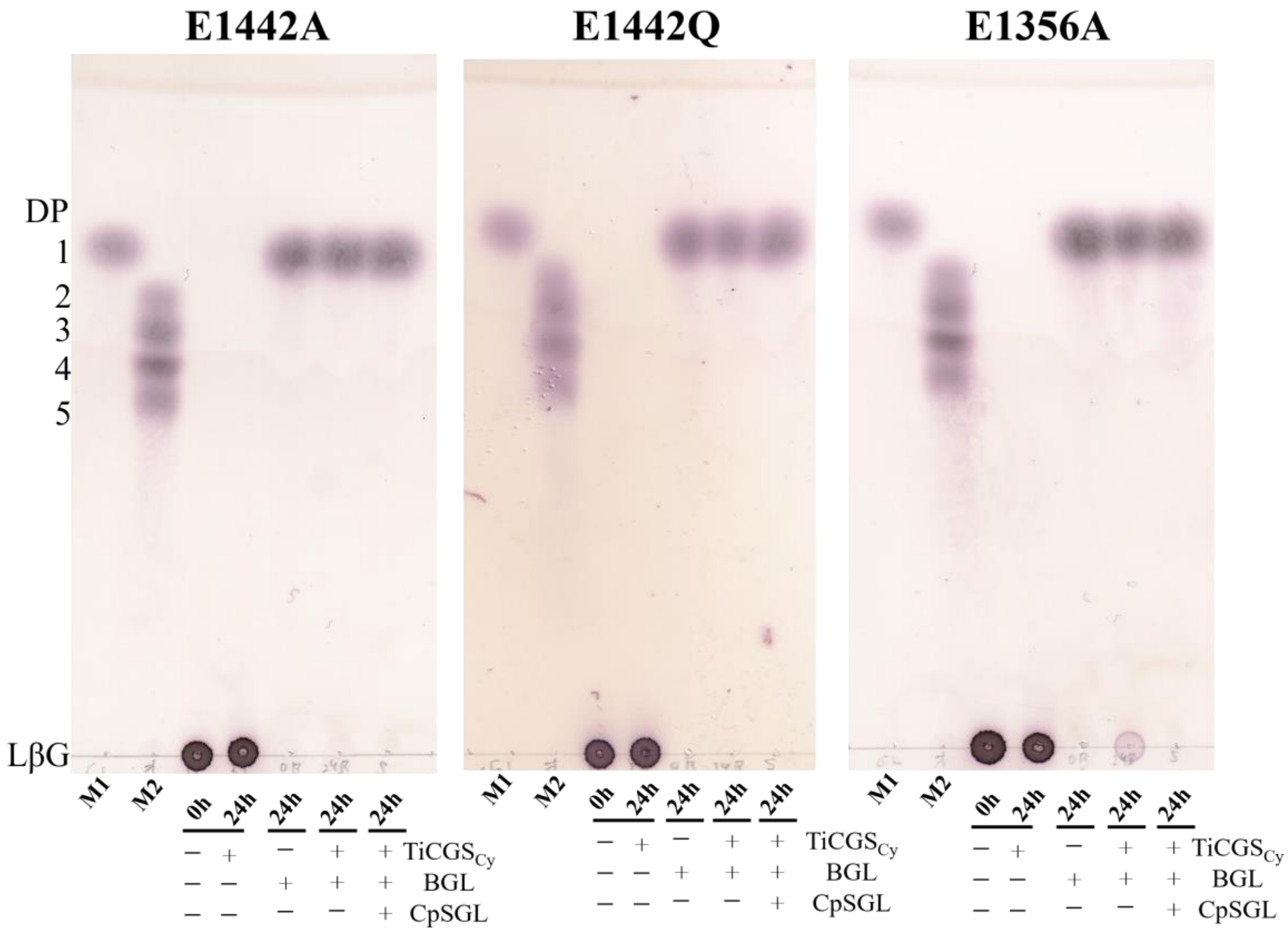

Fig. S8

TLC analyses of mutant TiCGS$_{Cy}$s. Lane M1, glucose (5 mM, 0.5 μl) was spotted. Lane M2, a 0.5 μl of mixture of Sop$_{2–5}$ (each 5 mM) was spotted. Horizontal lines on the TLC plates represent origins. Each sample (0.5–2 μl) was spotted on the origin.

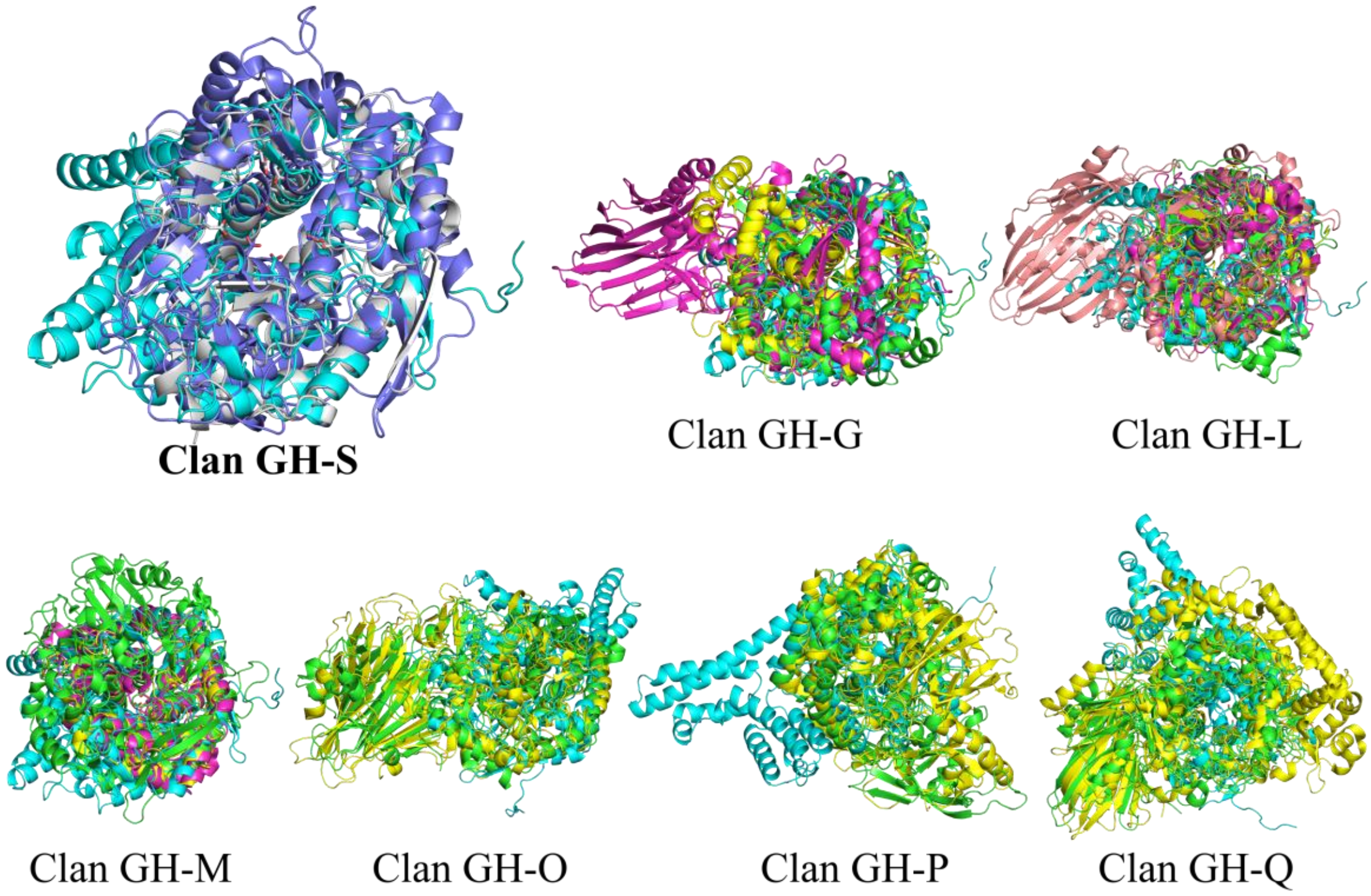

Fig. S9

Superimposition of the overall structure of TiCGS$_{Cy}$ (clan GH-S related) with those in each clan having the $(\alpha/\alpha)_6$ fold. The following structures are used for each clan: **clan GH-S related**: PDB IDs 8WY1 for TiCGS$_{Cy}$ (GH189), **clan GH-S**: PDB IDs 6IMW (GH162) (Tanaka et al. 2019), 5GZK (GH144) (Abe et al. 2017), **clan GH-G**: PDB IDs 2JG0 (GH37, yellow) (Gibson et al 2007), 5GOP (GH100, green) (Xie et al. 2016), 3D3I (GH63, magenta) (Kurakata et al. 2008), **clan GH-L**: PDB IDs 1GLM (GH15, green) (Aleshin et al. 1994), 1H54 (GH65, pink) (Egloff et al. 2001), 3QRY (GH125, magenta) (Gregg et al. 2011), 7QSJ (GH178, yellow) (Maranha et al. 2023), **clan GH-M**: PDB IDs 1KWF (GH8a, yellow) (Guérin et al. 2002), 1V5C (GH8b, magenta) (Adachi et al. 2004), 1FAE (GH48, green) (Parsiegla et al. 2000), **clan GH-O**: PDB

IDs 4C1O (GH52, green) (Espina et al. 2014), 5BVU (GH116, yellow) (Charoenwattanasatien et al. 2016), **clan GH-P**: PDB IDs 3WKX (GH127, green) (Ito et al. 2014), 6YQH (GH146, yellow) (McGregor et al. 2021), **clan GH-Q**: PDB IDs 1V7V (GH94, green) (Hidaka et al. 2004), 6HQ8 (GH149, yellow) (Kuhaudomlarp et al. 2019). The overall structures of TiCGS$_{Cy}$, TfSGL and CpSGL are shown in cyan, blue and white, respectively, while the other structures are shown in green, yellow and magenta.

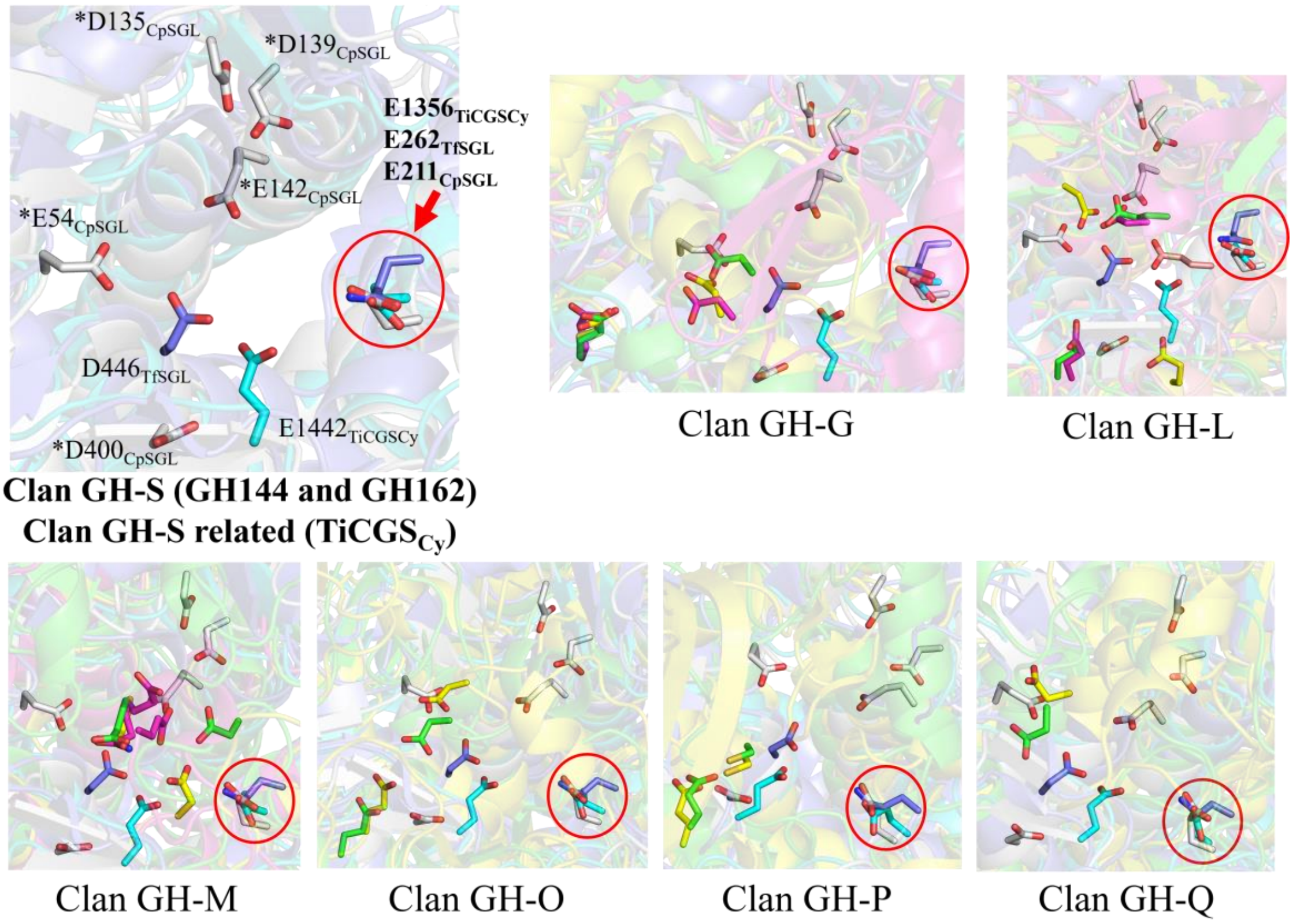

Fig. S10

Comparisons of catalytic residues between GH clans. Structures of clan GH-S, the clan GH-S related TiCGS$_{Cy}$, and a structure from each family which belongs to the compared GH clan having a $(\alpha/\alpha)_6$ fold are superimposed based on their overall structures. Their catalytic residues or candidate catalytic residues are represented by sticks. The catalytic residues and candidate catalytic residues of TiCGS$_{Cy}$, TfSGL and CpSGL are shown in cyan, blue and white, respectively, while those in other structures are shown in green, yellow and magenta. The catalytic residues whose positions are conserved within clan GH-S are circled in red. Colors of structures are consistent with Fig. S9.

References

Abe K, Nakajima M, Yamashita T, Matsunaga H, Kamisuki S, Nihira T, Takahashi Y, Sugimoto N, Miyanaga A, Nakai H, Arakawa T, Fushinobu S, Taguchi H (2017) Biochemical and structural analyses of a bacterial *endo*-β-1,2-glucanase reveal a new glycoside hydrolase family. J Biol Chem 292:7487–7506. https://doi.org/10.1074/jbc.M116.762724

Adachi W, Sakihama Y, Shimizu S, Sunami T, Fukazawa T, Suzuki M, Yatsunami R, Nakamura S, Takénaka A (2004) Crystal structure of family GH-8 chitosanase with subclass II specificity from *Bacillus* sp. K17. J Mol Biol 343:785–795. https://doi.org/10.1016/j.jmb.2004.08.028

Aleshin AE, Hoffman C, Firsov LM, Honzatko RB (1994) Refined crystal structures of glucoamylase from *Aspergillus awamori* var. *X100*. J Mol Biol 238:575–591. https://doi.org/10.1006/jmbi.1994.1316

Charoenwattanasatien R, Pengthaisong S, Breen I, Mutoh R, Sansenya S, Hua Y, Tankrathok A, Wu L, Songsiriritthigul C, Tanaka H, Williams SJ, Davies GJ, Kurisu G, Ketudat Cairns JR (2016) Bacterial β-Glucosidase Reveals the Structural and Functional Basis of Genetic Defects in Human Glucocerebrosidase 2 (GBA2). ACS Chem Biol 11:1891–1900. https://doi.org/10.1021/acschembio.6b00192

Egloff MP, Uppenberg J, Haalck L, van Tilbeurgh H (2001) Crystal structure of maltose

phosphorylase from *Lactobacillus brevis*: unexpected evolutionary relationship with glucoamylases. Structure 9:689–697. https://doi.org/10.1016/s0969-2126(01)00626-8

Espina G, Eley K, Pompidor G, Schneider TR, Crennell SJ, Danson MJ (2014) A novel β-xylosidase structure from *Geobacillus thermoglucosidasius*: the first crystal structure of a glycoside hydrolase family GH52 enzyme reveals unpredicted similarity to other glycoside hydrolase folds. Acta Crystallogr D Biol Crystallogr 70:1366–1374. https://doi.org/10.1107/S1399004714002788

Gibson RP, Gloster TM, Roberts S, Warren RAJ, Storch de Gracia I, García A, Chiara JL, Davies GJ (2007) Molecular basis for trehalase inhibition revealed by the structure of trehalase in complex with potent inhibitors. Angew Chem Int Ed Engl 46:4115–4119. https://doi.org/10.1002/anie.200604825

Gregg KJ, Zandberg WF, Hehemann JH, Whitworth GE, Deng L, Vocadlo DJ, Boraston AB (2011) Analysis of a new family of widely distributed metal-independent α-mannosidases provides unique insight into the processing of *N*-linked glycans. J Biol Chem 286:15586–15596. https://doi.org/10.1074/jbc.M111.223172

Guérin DMA, Lascombe MB, Costabel M, Souchon H, Lamzin V, Béguin P, Alzari PM (2002) Atomic (0.94 A) resolution structure of an inverting glycosidase in complex with substrate. J Mol Biol 316:1061–1069. https://doi.org/10.1006/jmbi.2001.5404

Guidolin LS, Ciocchini AE, De Iannino NI, Ugalde RA (2009) Functional mapping of *Brucella abortus* cyclic β-1,2-glucan synthase: identification of the protein domain required for cyclization. J Bacteriol 191:1230–1238. https://doi.org/10.1128/JB.01108-08

Hidaka M, Honda Y, Kitaoka M, Nirasawa S, Hayashi K, Wakagi T, Shoun H, Fushinobu S (2004) Chitobiose phosphorylase from *Vibrio proteolyticus*, a member of glycosyl transferase family 36, has a clan GH-L-like $(\alpha/\alpha)_6$ barrel fold. Structure 12:937–947. https://doi.org/10.1016/j.str.2004.03.027

Hisamatsu M, Amemura A, Harada T, Koizumi K, Utamura T, Okada Y (1984) Cyclic (1→2)-D-glucan produced by *Agrobacterium* and *Rhizobium*: the structure and the distribution of molecular weight. J Jpn Soc Starch Sci 31:117–123

Ito T, Saikawa K, Kim S, Fujita K, Ishiwata A, Kaeothip S, Arakawa T, Wakagi T, Beckham GT, Ito Y, Fushinobu S (2014) Crystal structure of glycoside hydrolase family 127 β-L-arabinofuranosidase from *Bifidobacterium longum*. Biochem Biophys Res Commun 447:32–37. https://doi.org/10.1016/j.bbrc.2014.03.096

Kuhaudomlarp S, Stevenson CEM, Lawson DM, Field RA (2019) The structure of a GH149 β-(1→3) glucan phosphorylase reveals a new surface oligosaccharide binding site and additional domains that are absent in the disaccharide-specific GH94 glucose-β-(1→3)-glucose (laminaribiose) phosphorylase. Proteins 87:885–892.

https://doi.org/10.1002/prot.25745

Kurakata Y, Uechi A, Yoshida H, Kamitori S, Sakano Y, Nishikawa A, Tonozuka T (2008) Structural insights into the substrate specificity and function of *Escherichia coli* K12 YgjK, a glucosidase belonging to the glycoside hydrolase family 63. J Mol Biol 381:116–128. https://doi.org/10.1016/j.jmb.2008.05.061

Maranha A, Costa M, Ripoll-Rozada J, Manso JA, Miranda V, Mendes VM, Manadas B, Macedo-Ribeiro S, Ventura MR, Pereira PJB, Empadinhas N (2023) Self-recycling and partially conservative replication of mycobacterial methylmannose polysaccharides. Commun Biol 6:108. https://doi.org/10.1038/s42003-023-04448-3

McGregor NGS, Coines J, Borlandelli V, Amaki S, Artola M, Nin-Hill A, Linzel D, Yamada C, Arakawa T, Ishiwata A, Ito Y, van der Marel GA, Codée JDC, Fushinobu S, Overkleeft HS, Rovira C, Davies GJ (2021) Cysteine Nucleophiles in Glycosidase Catalysis: Application of a Covalent β-L-Arabinofuranosidase Inhibitor. Angew Chem Int Ed Engl 60:5754–5758. https://doi.org/10.1002/anie.202013920

Nakajima M, Toyoizumi H, Abe K, Nakai H, Taguchi H, Kitaoka M (2014) 1,2-β-oligoglucan phosphorylase from *Listeria innocua*. PLOS ONE 9:e92353. https://doi.org/10.1371/journal.pone.0092353

Parsiegla G, Reverbel-Leroy C, Tardif C, Belaich JP, Driguez H, Haser R (2000) Crystal structures

of the cellulase Cel48F in complex with inhibitors and substrates give insights into its processive action. Biochemistry 39:11238–11246. https://doi.org/10.1021/bi001139p

Tanaka N, Nakajima M, Narukawa-Nara M, Matsunaga H, Kamisuki S, Aramasa H, Takahashi Y, Sugimoto N, Abe K, Terada T, Miyanaga A, Yamashita T, Sugawara F, Kamakura T, Komba S, Nakai H, Taguchi H (2019) Identification, characterization, and structural analyses of a fungal *endo*-β-1,2-glucanase reveal a new glycoside hydrolase family. J Biol Chem 294:7942–7965. https://doi.org/10.1074/jbc.RA118.007087

Xie J, Cai K, Hu HX, Jiang YL, Yang F, Hu PF, Cao DD, Li WF, Chen Y, Zhou CZ (2016) Structural Analysis of the Catalytic Mechanism and Substrate Specificity of *Anabaena* Alkaline Invertase InvA Reveals a Novel Glucosidase. J Biol Chem 291:25667–25677. https://doi.org/10.1074/jbc.M116.759290
